# Real-world outcomes of diffuse large B-cell lymphoma in the biosimilar era

**DOI:** 10.3389/fonc.2023.1248723

**Published:** 2023-10-03

**Authors:** Reena Nair, Gull Mohammad Bhat, Narendra Agrawal, Manju Sengar, Pankaj Malhotra, Soniya Nityanand, Chitra Lele, Pramod Reddy, Suresh Kankanwadi, Narendra Maharaj

**Affiliations:** ^1^ Department of Clinical Haematology, Tata Medical Centre, Kolkata, West Bengal, India; ^2^ Department of Medical Oncology, Sher-I-Kashmir Institute of Medical Sciences, Srinagar, Jammu & Kashmir, India; ^3^ Department of Hemato-Oncology & Bone Marrow Transplant, Rajiv Gandhi Cancer Institute and Research Center, Delhi, India; ^4^ Adult Hematolymphoid Disease Management Group, Medical Oncology, Tata Memorial Hospital, Mumbai, Maharashtra, India; ^5^ Department of Clinical Hematology & Medical Oncology, Post Graduate Institute of Medical Education and Research (PGIMER), Chandigarh, India; ^6^ Department of Hemotology, Sanjay Gandhi Post Graduate Institute of Medical Sciences, Lucknow, Uttar Pradesh, India; ^7^ ActuReal Services and Consulting Private Limited, Pune, Maharashtra, India; ^8^ Biologics, Dr. Reddy’s Laboratories Ltd, Bachupally, Hyderabad, India; ^9^ Biologics, Ex-Dr. Reddy’s Laboratories Ltd, Bachupally, Hyderabad, India

**Keywords:** rituximab, real world, DLBCL, biosimilar, Reditux™, Ristova^®^ Rituximab, Ristova^®^

## Abstract

**Background:**

Diffuse large B-cell lymphoma (DLBCL) is an aggressive and the most common type of non-Hodgkin lymphoma (NHL). The clinical use of rituximab has improved the treatment response and survival of patients with DLBCL. The introduction of rituximab biosimilar into healthcare system has helped in providing a cost-effective treatment to B-cell lymphoid malignancies as standard of care and has improved access to patients worldwide. The aim of this study was to observe the real-world effectiveness and safety of Reditux™ and Ristova^®^ in DLBCL patients.

**Methods:**

Observational study in adults with DLBCL receiving Reditux™ or Ristova^®^ across 29 centers in India (2015–2022). Effectiveness and safety were assessed up to 2 years after first dose.

**Results:**

Out of 1,365 patients considered for analysis, 1,250 (91.6%) were treated with Reditux™ and 115 (8.42%) with Ristova^®^. At 2 years, progression-free survival (PFS) 69% [hazard ratio (HR), 1.16; 95% CI, 0.80–1.67], overall survival (OS) 78.7% (HR, 1.20; 95% CI, 0.78–1.86), response rates, quality of life (QoL), and overall safety in both the cohorts were comparable. The best overall response rate (BORR) at 6 months was comparable with no statistically significant differences between the Reditux™ and the Ristova^®^ cohorts (89.2% vs. 94.3%). In multivariate analysis, BCL-2 and VAS were significant prognostic factors for PFS.

**Conclusion:**

Reditux™ and Ristova^®^ were comparable in real-world setting.

**Clinical Trial Registration:**

ISRCTN registry, identifier (ISRCTN13301166)

## Introduction

B-cell lymphoma represents 80%–85% of all non-Hodgkin lymphoma (NHL). Diffuse large B-cell lymphoma (DLBCL) accounts for 30%–40% of all B-cell lymphomas and is the commonest subtype in India (1, 2).

Rituximab (MabThera^®^/Rituxan^®^/Ristova^®^), a chimeric anti-CD20 monoclonal antibody has become standard of care for patients with DLBCL since its approval (3). It is given as the first-line treatment with cyclophosphamide, doxorubicin, vincristine, and prednisone (R-CHOP), as well as with other chemotherapeutic combinations. Administration of rituximab with CHOP therapy improves survival as compared to CHOP therapy alone (4, 5).

The introduction of rituximab biosimilars into healthcare systems has helped in providing a cost-effective treatment to B-cell lymphoid malignancies and has improved access to patients worldwide (6). Rituximab biosimilars are available at less than half the price of innovator in many countries (7, 8).

Reditux™ is a rituximab biosimilar launched by Dr. Reddy’s Laboratories in India in 2007 (9). Subsequently, it has been approved in nearly 26 other countries. It is structurally, physicochemically, analytically, and functionally similar to the innovator rituximab. Comparative clinical studies in DLBCL and rheumatoid arthritis patients have established the pharmacokinetic (PK) equivalence as well as safety, efficacy, and immunogenicity comparability of Reditux™ with MabThera^®^ (10, 11). Several other studies concluding similarity of safety and efficacy of Reditux™ have been published in both DLBCL and RA (12, 13).

Randomized, controlled, clinical studies enroll patients with uniform basal characteristics and have strict requirements for accurate interpretation of the results in order to address regulatory needs; consequently, they do not reflect real world patient populations. Non-interventional studies include patients with diverse baseline and disease characteristics from various clinical practice settings and offer an opportunity to evaluate drugs in a real-world setting (14). Real-world data from a prospective observational study (e.g., a registry that captures patient-level data on the use of the biosimilar and reference product) help in enhancing the confidence of medical practitioners, patients, regulators, and payers in the drug on interest (7, 15).

This observational study was designed to assess the effectiveness, safety, and impact on quality of life (QoL) of the DLBCL patients treated with Reditux™, as compared to those treated with innovator (Ristova^®^, the innovator rituximab, available in India), at 29 hospitals in India.

## Materials and methods

### Study design and methodology

A prospective, non-interventional registry study to observe the real-world effectiveness, safety, and impact on QoL of the patients who were started on with Reditux™ or Ristova^®^ as first-line treatment for previously untreated DLBCL in routine clinical practice was planned.

This study was conducted in accordance to requirements of the Declaration of Helsinki (October 2013) (16) and relevant National Laws and Regulations. The protocol and informed-consent forms were approved by Independent Ethics Committee at study centers. All patients provided their written informed consent. This registry also included patients who had already received their first dose with Reditux™ or Ristova^®^ identified retrospectively, after obtaining appropriate consent.

In accordance with routine clinic visits, data were collected every 6 months for a minimum of 2 years of follow-up. At the time of consent, information was collected about the patient characteristics [age, gender, Body mass index (BMI), educational level, and smoking status], ongoing treatment including previous dose(s) of rituximab and disease course. Every 6 months, data were collected on patients’ adverse events (AEs), outcomes, and survival. Information was collected on disease characteristics, rituximab infusion, use of chemotherapeutic medications, and occurrence of AEs. Each patient was also asked to complete the QoL instrument EuroQoL 5-dimension (EQ5D) at baseline, and every 6 months for 2 years. Patients who discontinued rituximab treatment after enrolment were also encouraged to continue participation up to 2 years for follow-up.

### Patients and treatment

Patients aged ≥ 18 years, previously untreated for DLBCL, who were on first-line treatment with either Reditux™ or Ristova^®^, irrespective of the dose, as part of combined chemotherapeutic treatment regimen were included in the study. DLBCL was classified on the basis of the WHO 2008 classification. Immunohistochemistry (IHC) was used for subtyping DLBCL into cell of origin (COO), as germinal center B cell (GCB) and non-GCB. IHC-based algorithm using biomarkers CD10, BCL-2, BCL-6, and MUM-1 was used for DLBCL subtyping (17, 18).

Patients treated in 29 both private and non-private hospitals (tertiary cancer centers, academic institutes, and government sponsored) under Oncology Care department were included in the study.

Based on the highest attained education, patients were categorized in to two groups, that is, higher education level group (graduation and above degrees) and lower education-level group (those who completed secondary education or below).

The study protocol did not include any instructions on rituximab product choice or any other patient management decisions.

### End points, study outcomes, and assessments

The primary end point was progression-free survival (PFS) at 2 years. Secondary end points included objective response rate (ORR), complete response (CR), partial response (PR), 2-year overall survival (OS), 2-year event-free survival (EFS), adverse drug reactions (ADRs), and QoL. According to the Revised Response Criteria for Malignant Lymphoma (19), an additional end point ORR was defined in the protocol as the sum of the rates of CR, and PR. Best overall response rate (BORR) was defined as the response rate evaluated on the basis of the best response, CR or PR, reported in the patient at any available time. BORR at 6 months was not defined in the protocol but was introduced *posteriori*. A time frame of 6 months was considered, because it represented the time where the response can more be convincingly attributed to the first line of treatment use to define inclusion in the study, and because most patients had response evaluation done within 181 days from first dose. Criteria for the overall response assessment were not defined in the protocol, and the assessment was open to investigator/site practice for patient management.

PFS was defined as the interval between the first rituximab infusion and the earliest date of disease progression (defined as a clinical diagnosis of relapse, progression, or refractory DLBCL) or death due to any cause. OS was defined as the interval from the first rituximab infusion until death from any cause. EFS was defined as the time from the first rituximab infusion until relapse or progression, unplanned re-treatment of lymphoma after initial immunochemotherapy, or death as a result of any cause.

Covariates determined at diagnosis included International Prognostic Index (IPI) risk category (low to low intermediate risk, 0–2 point; high-intermediate to high risk, 3–5 points), Eastern Cooperative Oncology Group Performance Status (ECOG PS), Ann Arbor stage of disease, Charlson Major Comorbidity Index **(**CCI), extra nodal sites, LDH level.

The effects of rituximab treatment on the QoL of patients were evaluated by the EQ-5D questionnaire and visual analogue scale (VAS). The VAS (EQ-5D) generates a single health status index by asking the patients to rate their current health by drawing a line from a box marked, “Your health state today” to the appropriate point on a 20-cm, 10-point VAS ranging from 0 (worst imaginable health) to 100 (best imaginable health) (20). The EQ-5D measures the five dimensions of mobility, self-care, usual activities, pain/discomfort, and anxiety/depression. Responses for each dimension range from 1 to 3, depending on whether the patient perceives (1) no problems, (2) some problems, or (3) major problems in that dimension of their health.

All AEs/SAEs and ADRs were monitored and reported throughout the entire course of the study. The AE reporting period began after the ICF sign off and continued through the post-treatment period till the end of the follow-up period. The assessment of severity was made irrespective of intervention relationship or seriousness of the event and was evaluated based on CTCAE Version 5.0 severity grading.

### Statistical analysis

The initial protocol planned a minimum sample size of about 2620 patients in an approximate ratio of 2:1 for Reditux™: innovator rituximab. In view of the lower than expected actual recruitment in the first 2 years of study, highly unequal distribution of patients in the two treatment arms in the real world setting and challenges of continued recruitment, the target sample size was revised to 2000 by an amended protocol.

Statistical analysis was performed to compare the cohorts who received either Ristova^®^ or Reditux™ using SAS^®^ version 9.4 for Windows. Descriptive statistics were used to characterize patient demographics, medical history, clinical characteristics, and treatment patterns including chemotherapeutic agents during the study period. Continuous variables were reported as “n,” mean (± standard deviation), median, and range. Categorical or ordinal variables were summarized as total counts, frequency, and proportion, and by subgroups where appropriate.

Fisher’s exact test or chi-square and t-test test were used for statistical comparisons. PFS, OS, and EFS were analyzed using log rank test and presented as Kaplan–Meier (KM) graphs. *P* < 0.05 were considered statistically significant (21), and differences were compared using the two-sided log rank test.

Multivariate analysis was performed to identify the impact of the prognostic factors on PFS the primary outcome, while adjusting for differences in confounding baseline variables, which are typical of observational study designs. Univariate analysis for one covariate at a time was done using logistic regression. The following confounding factors were considered for univariate analysis: age, Ann Arbor stage, BCL2, BMI, CCI, DLBCL_ABC *versus* DLBCL_GCB, ECOG, EDUCATION, IPI_ENODAL, IPI_INDEX, IPI_LDH, SMOKE, and VAS score.

Out of these confounders identified from univariate analysis, age, Ann Arbor stage, ECOG, and IPI_LDH were not used in the multivariate model. These variables are the components used to derive IPI_index, and IPI_index was used as one of the factors in the multivariate analysis. Rest of the confounders identified based on univariate logistic regression along with treatment group were used in multivariate logistic model. Backward selection technique was used to select covariates at 10% level of significance.

Multivariate analysis was performed using logistic regression analysis and backward elimination procedure. A significance level of 5% was used to select covariates to be retained in the final model. For all statistical analyses in the study, no imputation methods were used for missing values and missing data was not considered for any evaluations. Only available or collected patient data was used for primary and secondary analysis.

## Results

### Patient demographics

**Figure 1:**
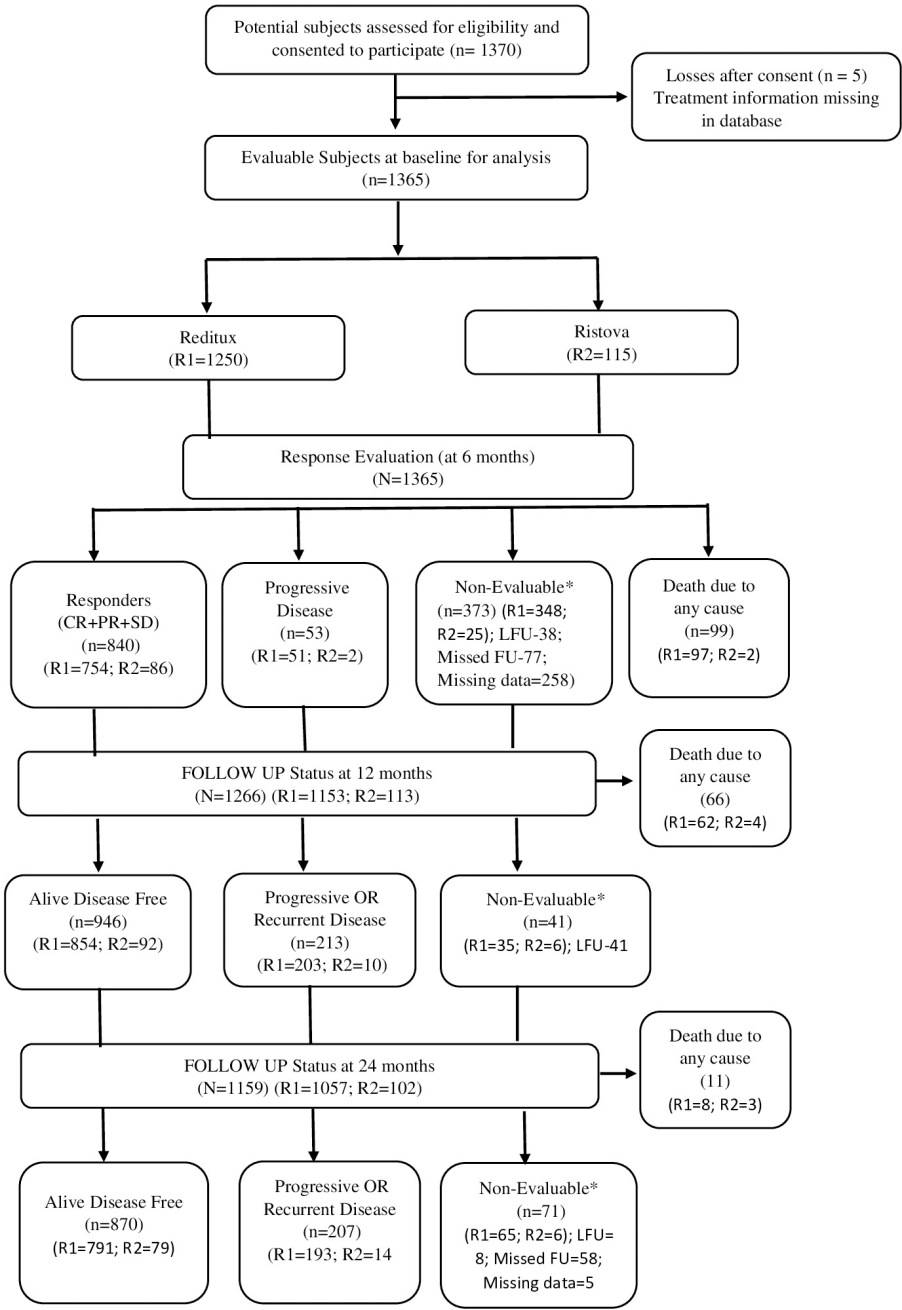
Patient disposition (all treated patients). The number of patients analyzed for each outcome is presented in the specific table/figure for the outcome. *Non-evaluable patients with missed follow-up (FU) were considered for analysis in subsequent follow-up visits if presented.

Between 25 March 2015 and 31 January 2019, 1,370 previously untreated DLBCL patients were assessed for eligibility, of which 1,365 participated in the study at 29 sites across 15 states and one union territory in India. Of these, 1,365 patients were treated with rituximab and were included in the analysis (data cutoff date 24 December 2019) and further evaluated at follow-up visits of intervals not longer than 6 months. The follow-up data until study completion in April 2022 is reported here. Of the 1,365 patients, 1,250 (91.6%) were treated with Reditux™ and 115 (8.4%) were treated with Ristova^®^ (**Figure 1**). Patient demographics and baseline clinical characteristics are summarized in **Table 1**.

**Table 1:** Patient demographics and baseline characteristics.

Characteristics	Reditux^TM^ (*N* = 1250)	Ristova^®^ (*N* = 115)	*p*-value
N	1250	115	
Age, years median (range)	55.1 (18.1, 88.5)	58.8 (20.0, 86.0)	0.0003*
Gender, *n* (%)			
Male	820 (65.6)	71(61.7)	
Female	430 (34.4)	44 (38.3)	0.4053**
Overall study duration, mean (SD) (year)	1.7 (0.9)	2.0 (0.9)	
Smoking status *n* (%)			
Current	66 (5.3)	2 (1.7)	
Past	209 (16.7)	14 (12.2)	0.0427**
Never	975 (78.0)	99 (86.1)	
Hospital type *n* (%)			
Private	420 (33.6)	77 (67.0)	
Non-private	829 (66.3)	38(33.0)	< 0.0001**
Missing	1 (0.1)	0 (0)	
Education level completed, *n* (%)			
Higher	460(37.6)	61 (62.2)	
Secondary (or below)	762 (62.4)	37 (37.8)	< 0.0001**
Missing	28 (2.2)	17 (14.8)	
*n*	1125	110	
BMI, kg/m^2^ (mean ± SD)	23.8 ± 4.68	25.8 ± 5.32	< 0.0001*
Duration of disease (months)			
n	1188	111	
Median months (range)	0.9 (0.0–68.0)	0.8 (0.1–3.4)	0.0003*
Mean months (SD)	1.3 (2.63)	0.9 (0.59)	
Diagnosis of DLBCL, *n* (%)	1014 (81.4)	99 (86.8)	0.5741**
NOS	882 (87.0)	85 (85.8)	
T-cell/histiocyte rich large B-cell lymphoma	93 (9.2)	12 (12.1)	
Primary mediastinal lymphoma	4 (0.4)	1 (1.0)	
Epstein-Barr virus (EBV)–positive DLBCL of the elderly	4 (0.4)	0	
Primary DLBCL of the central nervous system (CNS)	17 (1.7)	1 (1.0)	
Primary cutaneous DLBCL—leg type	11 (1.1)	0	
Intravascular large B-cell lymphoma	3 (0.3)	0	
IHC data subtypes			
*n* (%)	1098 (88.0)	106 (93.3)	
BCL2 (IHC)	506 (46.1)	53 (50.0)	
BCL6 (IHC)	571 (52.0)	52 (49.1)	
CD10 (IHC)(GCB)	388 (35.3)	45 (42.5)	
MUM1/IRF4 (IHC)(ABC)/NON-GCB	520 (47.4)	52 (49.1)	
MYC	32 (2.9)	9 (8.5)	
Activated B-cell (ABC)-DLBCL	300 (29.5)	25 (25.3)	
Germinal center B-cell (GCB)-DLBCL	339 (33.4)	33 (33.3)	
**n**	995	84	0.1279**
Normal LDH	459 (46.1)	46 (54.8)	
Elevated LDH	536 (53.9)	38 (45.2)	
**IPI age *n* (%)**			0.0126**
≤60	615 (62.0)	41 (48.2)	
>60	377 (38.0)	44 (51.8)	
**Ann Arbor stage *n* (%)**			0.7470**
Localized (stage I or II)	404 (40.6)	36 (42.4)	
Advanced (stage III or IV)	592 (59.4)	49 (57.6)	
**ECOG-PS: (0–4), *n* (%)**			0.0534**
Better performance (< 2)	698 (70.1)	68 (80.0)	
Worse performance (2–4)	298 (29.9)	17 (20.0)	
Missing	254 (20.3)	30 (26.1)	
**With more than one extra nodal site *n* (%)**			0.0125**
Yes	382 (38.4)	21 (24.7)	
Missing	254 (20.3)	30 (26.1)	
IPI score *n* (%)			0.5541**
Low to low intermediate risk group (IPI score, 0–2)	780 (65.4)	75 (68.2)	
High intermediate to high risk group (IPI score, 3–5)	413 (34.6)	35 (31.8)	
Missing	57(4.6)	5(4.3)	
**Charlson Major Comorbidity Index, *n* (%)**			0.0410**
Low CCI ( 0 or 1)	636 (50.9)	70 (60.9)	
Moderate to high CCI (2 or more)	613 (49.1)	45 (39.1)	
Missing	1 (0.1)	0	
Visual analog scale (EQ5D)			
n	1238	115	<0.0001*
Mean (SD)	63.4(19.0)	71.1(21.4)	
Median	60	70	
Missing	12(1.0)	0	

N = number of patients treated in each cohort.

n = number of evaluable patients for a particular characteristic.

BMI, body mass index; DLBCL, diffuse large B-cell lymphoma; NOS, not otherwise specified; BCL, B-cell lymphoma gene; CD, cluster of differentiation 10; MUM1/IRF4, multiple myeloma 1/interferon regulatory factor 4; IHC, immunohistochemistry; MYC, V-myc myelocytomatosis viral oncogene homolog; ABC, activated B cell; GCB, germinal center B cell; CNS, central nervous system; IPI, International Prognostic Index; LDH, lactate dehydrogenase; ECOG, Eastern Cooperative Oncology Group; EQ-5D, EuroQol 5 dimension.

*p-values are obtained using two sample t-test. **p-values are obtained using chi-square test or fisher exact test (for small sample size). ^Subjects may have multiple genomic data Subtypes and, hence, p-value and CIs are not displayed.

There were no significant differences between the two cohorts in terms of gender distribution, proportion of patients with elevated LDH level, IPI, and the distribution of stages or COO. Patients having current and past smoking status, presence of more than one extra nodal sites, treated at non-private hospital type and secondary (and below) education level were higher in proportion in Reditux™ cohort.

All DLBCL treatments were recorded at baseline including combination chemotherapy (CHOP-R and CHOP-like therapies including Rituximab), rituximab monotherapy, and others including immune modulators and targeted agents to evaluate their potential association on the outcomes of interest. Out of 1,099 (87.9%) eligible patients on DLBCL chemotherapy, 684 (62.2%) on Reditux, and out of 112 (97.4%) eligible subjects, 64 (57.1%) on Ristova^®^ received the CHOP-R standard therapy; 50 (4.6%) on Reditux and 2 (1.8%) on Ristova received CHOP-like therapies with rituximab and 17 patients were on rituximab monotherapy [15(1.4%) in Reditux and two (1.8%) in Ristova]. Approximately 30%–40% patients were on chemotherapies other than the above included immune modulators and targeted agents such as lenalidomide and ibrutinib (**Supplementary Table S1**).

The median (range) age of patients was 55.1 (18.1, 88.5) years in the Reditux™ and 58.8 (20.0, 86.0) years in the Ristova^®^ cohort. Of total patients, 65.6% were male in Reditux™ and 61.7% male in Ristova^®^ cohort. The mean duration of disease [1.3 (2.63) months and 0.9 (0.59) months since diagnosis in the Reditux^™^ and Ristova^®^ cohorts, respectively] was significantly higher in Reditux^™^. The proportion of patients who were current or past smokers was higher in the Reditux™ (5.3% current and 16.7% past smokers) as compared to the Ristova^®^ cohort (1.7% current and 12.2% past smokers) (**Table 1**).

The majority of patients from Ristova^®^ cohort were being treated at private hospitals (67%), whereas the majority of patients from Reditux™ cohort were being treated at non-private hospitals (66.3%) (**Table 1**). Approximately 62.2% of Ristova^®^-treated patients had acquired higher education (graduation) compared to 37.6% of Reditux™-treated patients.

The DLBCL subtype distribution is provided in **Table 1**. Approximately 60% of total patients in both the cohorts were classified as either the ABC type of DLBCL or GCB type of DLBCL. The proportion of patients expressing BCL-2 and BCL-6 proteins were 46.1% (*n* = 506) and 52% (*n* = 571), respectively, in the Reditux™ cohort and were 50% (*n* = 53) and 49.1% (*n* = 52), respectively, in the Ristova^®^ cohort.

The proportion of subjects with age >60 years at the time of IPI collection was significantly greater in the Ristova^®^ arm (51.8%) compared to the Reditux^™^ arm (38.0%). There were no apparent differences in the distribution of patients with Ann Arbor stages I/II among cohorts (40.6% in Reditux™ and 42.4% in Ristova^®^) and III/IV (59.4% in Reditux™ and 57.6% in Ristova^®^).

At baseline, 70.1% patients receiving Reditux and 80.0% receiving Ristova had an ECOG PS < 2.

Patients with more than one extra nodal site were 38.4% in Reditux™ and 24.7% in Ristova^®^ at baseline. The IPI prognostic index score did not differ between cohorts, the majority being in the good risk group (0–2). The serum LDH was ≥ upper limit normal (ULN) in 53.9% of the Reditux™ and in 45.2% of the Ristova^®^ cohort. Median VAS score was better in Ristova^®^ (70.0) compared to Reditux™ (60.0) (**Table 1**). More than half of the patients from Reditux™ (50.9%) and Ristova^®^ (60.9%) cohorts were in the low Charlson Comorbidity Index (CCI) group (0 or 1).

### Outcomes

#### Survival and prognostic factor

Of 1,256 evaluable patients who contributed for PFS data at 2 years, that is, those who were followed up for 2 year or had an event, 355 of 1,146 (31.0%) in the Reditux**™** cohort and 31 of 110 (28.2%) in the Ristova**^®^** cohort had progression of disease at 2 years. The estimated 2-year PFS-rates in the Reditux**™** and Ristova**^®^** cohort were 69.0% and 71.8%, respectively (**Table 2**).

**Table 2:** Efficacy outcomes.

Outcome	Reditux™ (*N* = 1250)	Ristova^®^ (*N* = 115)	HR/difference in PFS or OS (95% CI)	*p*-value
Progression-free survival at 2 years				
N	1146	110		
Progression event *n* (%)	355 (31.0)	31 (28.2)		
PFS rate (%)	(69.0)	(71.8)		
Median progression-free survival, months (95% CI)	NE	NE	HR = 1.16 (0.80, 1.67)	0.4348*
2-year PFS incidence rate per 100 patient years	30.18	25.64		
Difference in 2-year survival rate			Difference in PFS = 4.54 (−5.67, 14.75)	0.3836**
Overall survival at 2 years				
N	1250	115		
Deaths n (%)	266 (21.3)	22 (19.1)		
Median overall survival, months (95% CI)	NE	NE	HR = 1.20 (0.78, 1.86)	0.4065*
2-year overall survival rate per 100 patients years	14.41	11.85		
Difference in 2-year survival rate			Difference in OS = 2.56 (−3.12,8.25)	0.3764**
Event free survival at 2 years				
N	1244	114		
Events n (%)	511 (41.1)	42 (36.8)		
Median event free survival, months (95% CI)	NE	NE	HR = 1.22 (0.89, 1.68)	0.2087*
2-year event free survival rate per 100 patient-year	29.52	23.87		
Difference in 2-year event free survival rate			Difference in EFS = 5.65 (−2.70,14.01)	0.1845**
Best response rate				
n (%)	805 (64.4)	88 (76.5)		
BORR within first 6 months	718 (89.2)	83 (94.3)	Difference in proportion −5.13 (−10.42, 0.17)	0.1332***
Complete remission	470 (58.4)	49 (55.7)		
Partial remission	248 (30.8)	34 (38.6)		
Stable disease	36 (4.5)	3 (3.4)		
Progressive disease	51 (6.3)	2 (2.3)		

N = Number of patients treated in each cohort.

n = Number of evaluable patients for a particular outcome.

*The p-value is obtained using Log-rank test.

**The p-value (two-tailed) is obtained using z score test.

***The p-value is obtained using chi-square test (for small sample size, Fisher’s exact test is used).

Percentages are based on number of subjects in each treatment group at baseline as denominator.

CI, confidence interval; HR, hazard ratio.

**Figure 2:**
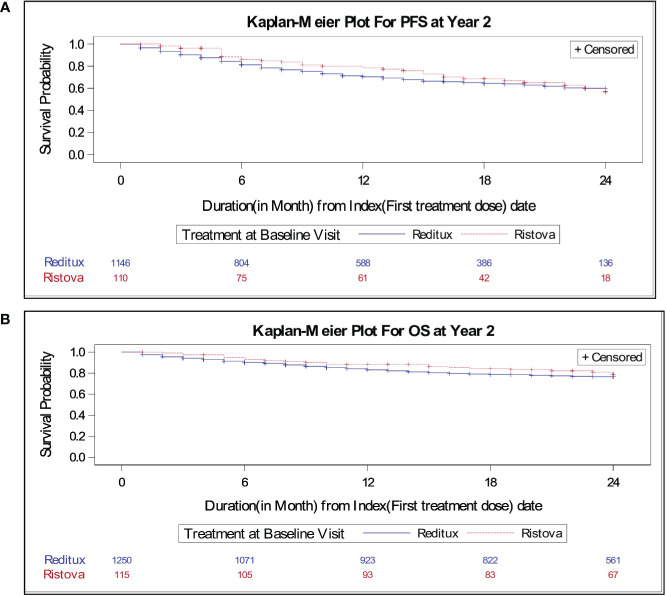
Kaplan–Meier estimates of **(A)** progression-free survival for patients with DLBCL treated with Reditux^™^ (*n* = 1146) and Ristova^®^ (*n* = 110) and **(B)** overall survival for patients with DLBCL treated with Reditux^™^ (*n* = 1250) and Ristova^®^ (*n* = 115).

The median PFS was not reached in patients in any cohort at the time of this analysis. There was no statistically significant difference in PFS between the two cohorts [hazard ratio (HR), 1.16; 95% CI, 0.80–1.67] (**Table 2**). KM curves for PFS for both cohorts overlap after 20 months, suggesting a comparable PFS in both cohorts (**Figure 2A**). Based on KM curve, estimated 2-year PFS was approximately 70% for both cohorts (**Figure 2A**).

Of 1,358 evaluable patients for EFS, 511 of 1,244 (41.1%) in Reditux**™** cohort and 42 of 114 (36.8%) patients in the Ristova**^®^** cohort had a relapse/progression or unplanned re-treatment of lymphoma after the initial treatment or died within 2 years. There was no statistically significant difference in EFS between the two cohorts (HR, 1.22; 95% CI, 0.89–1.68) (**Table 2**).

Of the 1,365 evaluable patients assessed for OS, 266 of 1,250 (21.3%) on Reditux**™** and 22 of 115 (19.1%) patients on Ristova**^®^** cohort met an event within 2 years. No significant difference was observed between cohorts in terms of overall survival at 2 years (HR, 1.20; 95% CI, 0.78–1.86). The 2-year overall survival rate per 100 patient years was comparable between the cohorts (14.41 vs. 11.85) (**Table 2**). The KM curves for OS overlap at 24 months, suggesting a comparable OS in both cohorts. Two-year OS was around 80% for both cohorts (**Figure 2B**). The incident rates per 100 person years at risk having an event of death within 2 years was calculated and compared between treatments. Time at risk was defined as the interval between the date of the first infusion of treatment to the date of death or end of 2 years (or until study completion, loss to follow-up, withdrawal from the study, whichever occurs first). Two-year survival incidence rate per 100 patient years was not significantly different between cohorts (IR, 2.56; 95% CI, −3.12– 8.25).

Univariate analysis showed that better VAS score (*p* = 0.002), lower IPI (0–2) (*p* = 0.0004), absence of BCL-2 expression (*p* = 0.0158), Ann Arbor localized stages I–II (*p* = 0.0006), DLBCL_ABC versus DLBCL_GCB (*p* = 0.0422), no IPI_ENODAL (*p* = 0.0026), no IPI_LDH (*p* = 0.0032), age **≤**60 (*p* < 0.001), and ECOG **<**2 (*p* = 0.0010) to be associated with longer PFS. The administered treatment (Reditux**™** or Ristova**^®^**) was not a statistically significant predictor of PFS at 2 years neither in univariate nor in multivariate analysis (**Table 3**).

**Table 3:** Multivariate analysis of possible prognostic factors impacting PFS at 2-year outcome.

Covariates	PFS at 2 years
	OR (95% CI)
Analysis using significant confounding factors	
BCL-2 No vs. Yes BMI IPI_INDEX 0–2 vs. 3–5	0.5939 (0.4004–0.8809) 0.9629 (0.9263, 1.0009) 0.7140 (0.5003, 1.0189)
Baseline treatment Reditux™ vs. Ristova^®^	1.4096 (0.7664–2.5925)
Model with confounders: TREATMENT BCL2 BMI IPI_INDEX	
Analysis excluding significant confounding factors (without BCL2 and DIAG_DLBCL_R)	
VAS	0.9897 (0.9829–0.9965)
IPI index 0–2 vs. 3–5	0.6769 (0.5210–0.8794)
Education higher vs. rest	0.8141 (0.6266–1.0577)
Baseline treatment Reditux™ vs Ristova^®^ Model with confounders: TREATMENT EDUCATION IPI_INDEXX VAS	1.0130 (0.6263–1.6385)

BCL-2, B-cell lymphoma 2 gene; VAS, Visual analog scale; IPI, International Prognostic Index; DIAG_DLBCL_R, DLBCL subtype classification- (ABC vs GCB).

With inclusion of significant confounding factors in multivariate analysis, odds ratio (OR = 0.5939; 95% CI, 0.4004–0.8809) indicated a known prognostic clinical characteristic (BCL2), as the most significant prognostic factor of PFS at 2 years (**Table 3**).

After excluding significant confounding factors (without BCL2 and ABC vs. GCB subtype), which may be concealing other substantial factors affecting the PFS at 2 years in multivariate analysis, better VAS score (OR = 0.9897; 95% CI, 0.9829–0.9965) turned to be an independent favorable prognostic factor for PFS at 2 years (**Table 3**). As IPI index is based on five independent factors, which also included age and ECOG, these (age and ECOG) were not considered although they were significant predictors in univariate analysis. Only inclusive IPI index score was considered in multivariate analysis.

#### Responses

In the Reditux™ cohort, 58.4% patients achieved complete remission (CR) and 55.7% in the Ristova^®^ cohort. In total, 30.8% patients in the Reditux™ cohort and 38.6% patients in the Ristova^®^ cohort achieved partial remission (PR) and 4.5% patients in the Reditux™ and 3.4% patients in the Ristova^®^ cohort had stable disease (SD) (**Table 2**). A total of 893 subjects (805 in Reditux^™^ and 88 in Ristova^®^) had evaluable data to estimate BORR within first 6 months from treatment start. The BORR at 6 months was comparable with no statistically significant differences between the Reditux™ and the Ristova^®^ cohorts (89.2% vs. 94.3%) (difference in the proportion −5.13; 95% CI, (−10.42, 0.17); *p* = 0.1332).

An exploratory descriptive analysis of best response rate in DLBCL patients categorized based on Ann Arbor stages was carried out. There was no statistically significant difference between the treatment groups with respect to Ann Arbor stage. Patients with DLBCL stage I or II disease with CR/PR in Reditux™ (*n* = 283, 31.6%) compared to Ristova^®^ (*n* = 29, 30.9%) were also found to be comparable. Patients with DLBCL stage III or IV disease had comparable CR/PR in Reditux™ (*n* = 392, 43.8%) as compared to Ristova^®^ (*n* = 40, 42.6%). A similar trend was observed in the rates of progression free and overall survival (**Table 4**). The mean age [55.8 (SD = 15.33)] in patients with progressive disease was higher than the patients who did not have progressive disease [51.7 (SD = 14.58)] in the Reditux™ cohort. Similar findings were observed with progressive [56.87 (SD = 16.95)] and non-progressive disease [57.80 (SD = 13.62)] in the Ristova^®^ cohort (**Supplementary Table S3**).

**Table 4:** Comparison of treatment groups with respect to Ann Arbor staging [(stage 1 or stage II) versus (stage III or IV)] for each survival outcomes.

	Reditux (*N* = 1250) *n* (%)	Ristova (*N* = 115) *n* (%)	Difference in percentage (95% CI)	*p*-value**
BORR				
Number of subjects excluded from analysis*	354 (28.3)	21 (18.3)		
Number of subjects used in analysis* (N1)	896 (71.7)	94 (81.7)		
DLBCL staging for subjects with CR/PR				
Ann Arbor Stage	801 (89.4)	87 (92.6)		0.9868
Stage I or II	283 (31.6)	29 (30.9)	0.73 (−9.09, 10.55)	
Stage III or IV	392 (43.8)	40 (42.6)	1.20 (−9.31, 11.71)	
Missing	126 (14.1)	18 (19.1)		
DLBCL staging for subjects with SD/PD				
Ann Arbor Stage	95 (10.6)	7 (7.4)		0.5942
Stage I or II	23 (2.6)	2 (2.1)	0.44 (−2.66, 3.53)	
Stage III or IV	49 (5.5)	2 (2.1)	3.34 (0.07, 6.62)	
Missing	23 (2.6)	3 (3.2)		
PFS				
Number of subjects excluded from analysis*	104 (8.3)	5 (4.3)		
Number of subjects used in analysis* (N1)	1146 (91.7)	110 (95.7)		
DLBCL staging for subjects with progression				
Ann Arbor stage	355 (31.0)	31 (28.2)		0.7748
Stage I or II	88 (7.7)	8 (7.3)	0.41 (−4.69, 5.50)	
Stage III or IV	188 (16.4)	15 (13.6)	2.77 (−3.99, 9.53)	
Missing	79 (6.9)	8 (7.3)		
DLBCL staging for subjects without progression				
Ann Arbor stage	791 (69.0)	79 (71.8)		0.8395
Stage I or II	285 (24.9)	27 (24.5)	0.32 (−8.10, 8.75)	
Stage III or IV	368 (32.1)	33 (30.0)	2.11 (−6.87, 11.09)	
Missing	138 (12.0)	19 (17.3)		
OS				
Number of subjects excluded from analysis*	0	0		
Number of subjects used in analysis* (N1)	1250 (100.0)	115(100.0)		
DLBCL staging for subjects with event				
Ann Arbor stage	266 (21.3)	22 (19.1)		0.9999
Stage I or II	64 (5.1)	5 (4.3)	0.77 (−3.15, 4.69)	
Stage III or IV	143 (11.4)	10 (8.7)	2.74 (−2.70, 8.19)	
Missing	59 (4.7)	7 (6.1)		
DLBCL staging for subjects without event				
Ann Arbor stage	984 (78.7)	93 (80.9)		0.8468
Stage I or II	340 (27.2)	31 (27.0)	0.24 (−8.23, 8.72)	
Stage III or IV	449 (35.9)	39 (33.9)	2.01 (−7.05, 11.06)	
Missing	195 (15.6)	23 (20.0)		

• Best Overall Response Rate (BORR) represents subject achieved either a complete response (CR) or a partial response (PR), a best available response is selected for a subject from all the available responses at all follow-ups till datacut.

• For overall survival, death is an event.

Percentages are based on number of subjects used in analysis (N1) as denominator.

*Percentages are based on number of subjects in each treatment group (N) at baseline as denominator.

**p-value is obtained using chi-square test (for small sample size, Fisher’s exact test is used).

#### EuroQoL five-dimension 3 level and visual analog scale score

**Figure 3:**
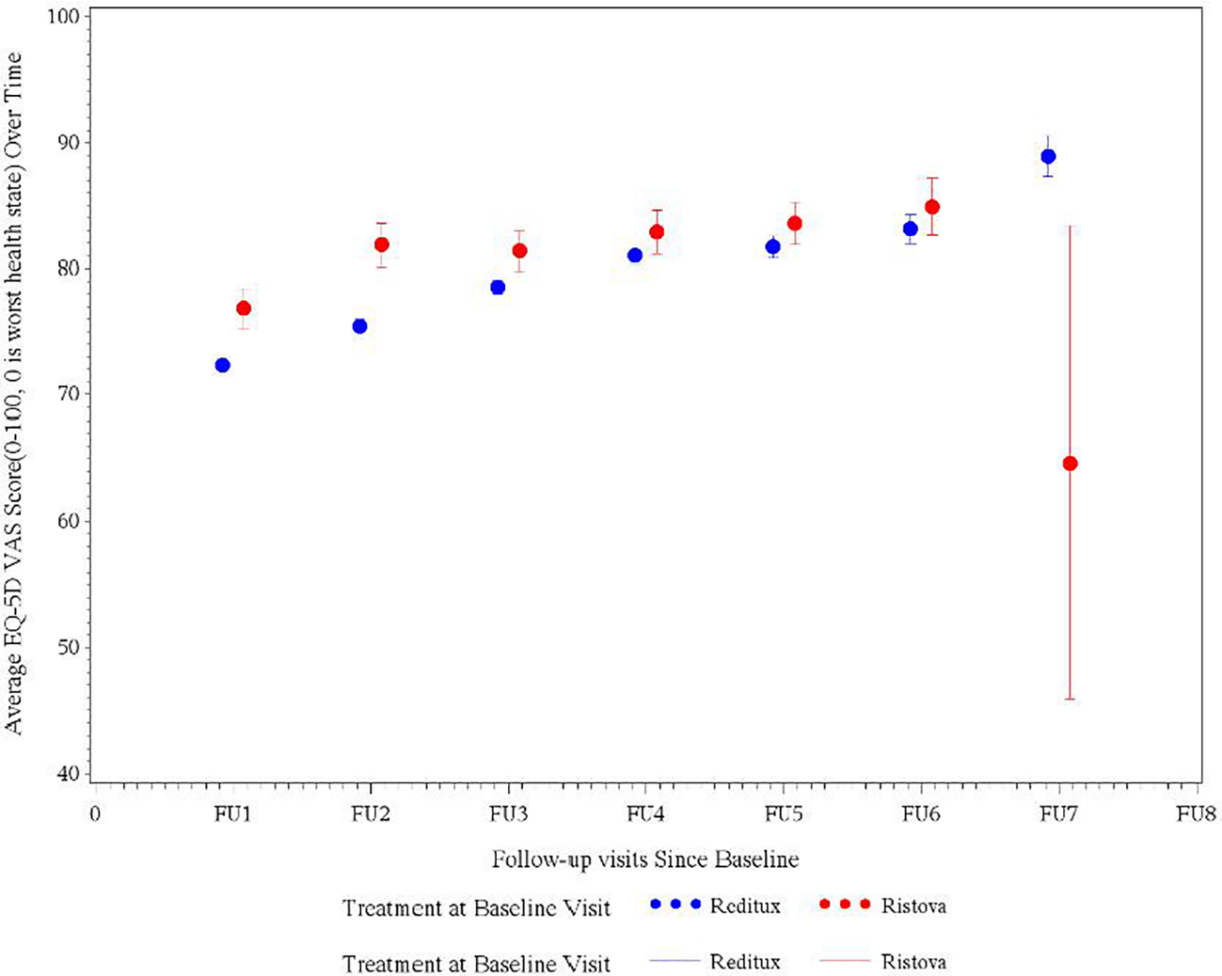
Average EQ5D quality of life visual analog scale (VAS) score measured at different follow-up visits in DLBCL patients in the Reditux™ and Ristova^®^ cohorts.

At baseline, the mean VAS score for the Reditux™ cohort was 63.6 (19.00) and for Ristova^®^ was 70.9 (21.62) points. From the baseline, a significant improvement of 16.0 (20.37) (*p* < 0.0196) points in mean VAS score was observed in the Reditux™ cohort as compared to a change of 8.3 (26.67) in the Ristova^®^ cohort after 2 years of treatment (**Table 5**). After 2 years of treatment at follow-up visit 4, mean VAS score was 81.0 (13.77) in Reditux™-treated patients compared to 82.9 (14.61) in Ristova^®^ cohort (**Table 5** and **Figure 3**).

**Table 5:** VAS score (EQ-5D).

	Reditux™		Ristova^®^		*p*-value for change
	Value	Change from baseline	Value	Change from baseline	
Baseline					
N	1250		115		
n	1237		114		
Mean (SD) VAS score	63.6 (19.00)	NA	70.9 (21.62)	NA	NA
2 years					
N	767	767	79	79	
n	710	702	71	71	
Mean (SD) VAS score	81.0 (13.77)	16.0 (20.37)	82.9 (14.61)	8.3 (26.67)	< 0.0196*

N = number of subjects in each at each follow-up visit.

n = Number of subject with non missing QOL data at that follow-up visit.

*p-values are obtained using two sample t-test.

EQ-5D, EuroQoL 5 dimension; NA, not applicable.

**Figure 4:**
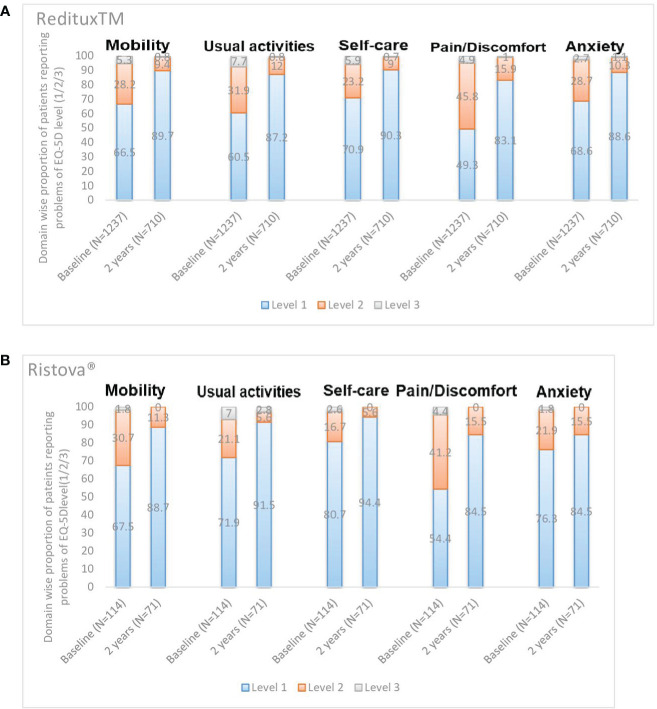
Domain wise proportion of patients reporting problems of EQ-5D level (1/2/3) at baseline and at 2 years. **(A)** Reditux™ and **(B)** Ristova^®^.

After 2 years of treatment, most patients reported no problems (Level 1) with five dimensions of mobility (89.7% in Reditux™ vs. 88.7% in Ristova^®^), self-care (90.3% vs. 94.4%), performing their usual activities (87.2% vs. 91.5%), and no pain/discomfort (83.1% vs. 84.5%) or anxiety/depression (88.6% vs. 84.5%). Proportion (varying from 83.1% to 94.4%) of patients reporting no problems (Level 1) in each dimension increased after 2 years of treatment with an apparent improvement in Reditux™ compared to Ristova^®^ cohort (**Figure 4**).

##### Safety

A total of 244 AEs (Reditux™ = 216 and Ristova^®^ = 28) were reported in 109 patients [Reditux™ = 95 (7.6%) and Ristova^®^ = 14 (12.2%)]. In total, 6.2% patients (77 of 1,250) reported at least one serious AE in Reditux™, whereas 7.8% patients (9 of 115) reported at least one serious AE in Ristova^®^. A total of 47 (3.8%) subjects in the Reditux^™^ group experienced AEs that were related to the study treatment compared to four (3.5%) patients in the Ristova^®^ group (**Table 6**).

**Table 6:** Overview of safety outcomes.

Variable	Reditux^TM^ (*N* = 1250)	Ristova^®^ (*N* = 115)	Total (*N* = 1365)
Adverse Events (AEs), n	216	28	244
Number of patients with at least One AE *n* (%)	95 (7.6%)	14 (12.2%)	109 (8.0%)
Number of patients with at least One serious AE *n* (%)	77 (6.2%)	9 (7.8%)	86 (6.3%)
Patients with AEs by severity			
Mild	28 (2.2%)	10 (8.7%)	38 (2.8%)
Moderate	23 (1.8%)	3 (2.6%)	26 (1.9%)
Severe	17 (1.4%)	5 (4.3%)	22 (1.6%)
Life threatening	5 (0.4%)	0	5 (0.4%)
Death	37 (3.0%)	2 (1.7%)	39 (2.9%)
Number of treatment-related adverse events (trAE)	117	8	125
Number of subjects with at least one AE	47 (3.8%)	4 (3.5%)	51 (3.7%)
Number of subjects with at least one serious AE	35 (2.8%)	4 (3.5%)	39 (2.9%)
Subjects with AE by severity			
Mild	15 (1.2%)	3 (2.6%)	18 (1.3%)
Moderate	13 (1.0%)	1 (0.9%)	14 (1.0%)
Severe	14 (1.1%)	2 (1.7%)	16 (1.2%)
Life threatening	4 (0.3%)	0	4 (0.3%)
Death	9 (0.7%)	1 (0.9%)	10 (0.7%)

Percentages are calculated using number of subjects in each treatment group at baseline as a denominator.

N = number of patients treated in each cohort.

n = number.

trAE, treatment-related adverse event.

In total, 39 patients [37 (3.0%) in the Reditux™ and 2 (1.7%) in the Ristova^®^ cohort] died during the study. From the reported deaths, nine (0.7%) in the Reditux™ and one (0.9%) in the Ristova^®^ were assessed as treatment related. The infusion related AEs for Reditux™ [*n* = 11; (0.9%)] were collected but were missing for Ristova^®^.

The percentage of patients who experienced at least one treatment-related AE (3.8% vs. 3.5%), at least one treatment-related serious AE (2.8% vs. 3.5%) and severe AEs (1.1% vs. 1.7%) did not differ much between the Reditux™ and Ristova^®^ cohorts (**Table 6**). The frequency, type, and severity of treatment-related AEs were comparable between the cohorts. Treatment-related Grade 3 AEs for patients in the Reditux cohort were febrile neutropenia (five patients); neutropenia and infusion-related reaction (three patients each); pyrexia and mucosal inflammation (two patients each); and left ventricular dysfunction, bacterial sepsis, septic shock, decreased appetite, and tumor lysis syndrome (one patient each). Treatment-related severe AEs for patients in the Ristova cohort were constipation and interstitial lung disease (one patient each). Treatment-related Grade 4 events for patients in the Reditux cohort were febrile neutropenia and hyponatremia (two patients each), neutropenia, hypertension, and tachycardia (one patient each). No subjects had Grade 4 events in the Ristova cohort (**Supplementary Table S4**). No new signals were identified during this post marketing observational study.

## Discussion

The clinical use of rituximab has improved the treatment response and survival of patients with DLBCL. Reditux was found to be highly similar to rituximab reference medicinal product based on a comprehensive totality of evidence with no clinically meaningful differences in safety and efficacy (10, 11, 22). Nonetheless, only limited comparative data of Reditux™ with innovator rituximab in a real-world scenario is available (13, 23, 24).

After the anti-CD20 monoclonal antibody rituximab was launched in the market, the survival of patients with B-cell NHL has improved, but rituximab remained out of reach and underutilized in low- and middle-income nations for more than a decade. Only 35% of patients with DLBCL could afford rituximab in 2010. This resulted in a 20% drop in 2-year overall survival compared to individuals who received rituximab (6). Ten years later, rituximab is given to 95% of DLBCL patients as standard of care. This, much-needed improvement mainly resulted from the availability of rituximab biosimilars together with several promotional initiatives in India to promote patient access (6).

We observed that the proportion of Reditux™ (*n* = 1250; 91.6%) prescriptions were many folds higher, as compared to Ristova^®^ (*n* = 115; 8.42%) in the study. We acknowledge this unequal prescription distribution as an expected real-world scenario in low/middle income countries due to the need of treating patients with affordable drugs, which should be taken in to consideration while interpreting study findings (25, 26). With no influence on physicians’ prescribing decisions, in observational studies, the reasons for prescribing Reditux™ or Ristova^®^ to patients were not recorded. However, we speculate that the high number of Reditux™ prescriptions noted during the study might have been influenced by its established widespread clinical use of rituximab over several years, which in turn might have enhanced the confidence of medical practitioners in the safety and efficacy of Reditux™ in their routine practice ever since its availability in India. The cost difference between two treatments and, hence, affordability might also have played a role in treatment selection. High number of Reditux™ prescriptions indicate global acceptance of biosimilar as observed in other published studies (12, 23, 27). A similar prescription pattern was noted in a real-world study conducted in Germany (2017–2019) where, within 2 years, the proportion of rituximab prescriptions for NHL/CLL for rituximab biosimilar increased from 12.0% to 83.0% against innovator rituximab with 90% of patients receiving a rituximab biosimilar by the end of the observation time (15). Another multi-country study involving 123 oncologists in Europe has reported that 95% of total 1,918 NHL patients switched from innovator rituximab to rituximab biosimilar within three months. The top three reasons for prescribing biosimilar rituximab to their NHL patients were “approved standard of care” (71%), “proven efficacy” (50%), and “on formulary/hospital policy” (32%) (28).

Due to aforementioned unequal distribution of patients in the two treatment arms, further analysis of sub-populations (patients with subtypes of lymphoma, patients receiving Rituximab with CHOP, etc.) was not performed as it was less likely to yield statistically meaningful results. Moreover, such an analysis of such sub-populations was not planned per protocol and in the statistical analysis plan. Survival for DLBCL patients has continued to improve in recent years with the advent of rituximab-based therapies. In the present study, median PFS was not reached in either cohort due to the relatively short follow-up period of 2 years. Two-year PFS rate (69.0% in the Reditux™ vs. 71.8% in the Ristova^®^) (HR, 1.16; 95% CI, 0.80–1.67) was not significantly different between two cohorts. These observations are in agreement with results from another real-world study in DLBCL patients treated with R-CHOP, where a PFS rate of 82% with no median PFS reached in the overall population (29). Despite the differences in study design and enrolled patients, the 2-year PFS rate [74·9% (95% CI 70.9–78.9] observed in a randomized control trial is comparable with the 2-year PFS observed in present study (30). As expected, patients with advanced DLBCL (Ann Arbor stage III or IV) progressed more frequently than patients with localized DLBCL (Ann Arbor stage I or II) in the Reditux™, which is consistent with what has been reported previously (31). The EFS (HR, 1.22; 95% CI, 0.89–1.68) was not significantly different between cohorts. The overall survival improved in both cohorts in a similar manner with 266 (21.3%) deaths in the Reditux™ and 22 (19.1%) deaths in the Ristova^®^ (HR, 1.20; 95% CI, 0.78–1.86) in the 2-year follow-up period. The KM estimate of time to event for OS for both cohorts overlapped after 24 months, suggesting a similar overall survival in both cohorts.

The Best ORR was considered to accommodate the inconsistent frequency of imaging evaluations in this observational study and has been used in other real-world studies as well as in clinical trials in first-line DLBCL (32). It was not defined in the protocol but BORR at 6 months was introduced *posteriori* as it is more suitable for use in the presence of incomplete information typical in observational studies. The BORR at 6 months in the Reditux™ cohort was [89.2% (CR, 58.4% and PR, 30.8%)] not significantly different to the Ristova^®^ cohort [94.3% (CR, 55.7% and PR, 38.6%)]. Another non-interventional study reports an ORR of 94.2% (CR, 55.0%; CRu, 18.2%; and PR, 20.9%) in R-CHOP treated DLBCL patients (33). A study involving 152 patients observed a similar ORR between Reditux™-CHOP and Ristova™ or Ikdgar™ (another rituximab biosimilar)-CHOP (13).

The univariate analysis identified that better VAS score, lower IPI score (0–2), ECOG <2, an absence of BCL-2 expression, DLBCL_ABC versus DLBCL_GCB, Ann Arbor stages I–II and age ≤60 were associated with longer PFS at 2 years and were possible favorable prognostic factors for PFS (all *p* < 0.1, which was the threshold used for inclusion of variables in multivariate analysis). Including these selected covariates in the multivariate analysis, it indicated that BCL-2 expression is the most significant independent prognostic factor for PFS, which is possibly owing to little patient data (50%) for this covariate. The prognostic value of BCL-2 protein in DLBCL is still controversial, reflecting the heterogeneity of the disease and different molecular techniques used (34, 35). One study suggested that BCL-2 expression was correlated with short survival in DLBCL patients. BCL-2 expression was found as predictive of survival in DLBCL in another study (36, 37). Therefore, after excluding BCL-2 and DLBCL subtype diagnosis (ABC vs. GCB) from the analysis, VAS was found to be independent favorable prognostic factors for PFS.

Health state and a perception of the good quality-of-life improved by 16.0 (SD = 20.37) points change in the mean EuroQoL-5D 3L VAS score after 2 years of Reditux™ therapy as compared to 8.3 (SD = 26.67) points change in Ristova^®^-treated cohort. These health benefits were also translated into a good perception of patient QoL, as reported by the EQ-5D 3 level questionnaire responses. Most patients from either cohort reported no problems (Level 1) with five dimensions of mobility, self-care, performing their usual activities, and no pain/discomfort or anxiety/depression after 2 years of treatment. The number of patients reporting no problems (Level 1) in each dimension improved more from baseline in the Reditux™ cohort compared to Ristova^®^ cohort. The reasons for the numerically greater improvement in the Reditux™ cohort are unclear.

The safety results were consistent with the known safety profile of rituximab, similar ADR frequencies and severity observed in DLBCL patients treated with either product (10–12). No infusion related AEs were reported for the Ristova^®^ cohort in the study. This appears to be a case of detection bias which is frequent in real word studies (38). For reference, the incidence of infusion related AEs reported in the patient information leaflet for Rituxan^®^ (rituximab) was highest during the first infusion (77%) and decreased with each subsequent infusion (39).

## Implications and limitations

By virtue of being a real world, uncontrolled, non-interventional study, this study supports the use of biosimilar rituximab in the management of DLBCLs, although our results should be interpreted in light of pertinent constraints. First, unequal distribution of patients between cohorts and unequal distribution of certain baseline characteristics that necessitates us to rely on cautious evaluation and adjustment of baseline imbalances by the use of multivariate statistical method. Second, missing values that are expected in a real-world study as was observed in an earlier study (27) could lead to the possibility of not detecting some variables as relevant predictors of response. In addition, higher number of patient dropouts was observed during the course of the study. The only logical explanation for the drop out of patients is the real world nature of the study and the natural course of the disease.

## Conclusion

Similar efficacy and safety between Reditux™ and Ristova^®^ in DLBCL patients were concluded in this large real-world study. Despite the small differences in demographic profile and baseline characteristics, the two treatments were comparable in terms of PFS at 2 years, EFS, BORR, OS, and safety at 2 years.

## Summary points

• This observational analysis suggests that treatment with either Redtux™ or Ristova^®^ both have a comparable real-world effectiveness, safety, and impact on QoL.
• Overall, the study included 1,370 DLBCL patients treated at 29 hospitals in India. In these real-world patients in clinical practice, the median PFS, EFS, and OS duration at the end of 2 years could not be estimated at the time of reporting these results. PFS ([HR], 1.16; 95% CI, 0.80–1.67), OS (HR, 1.20; 95% CI, 0.78–1.86), and response rates did not differ significantly between the two cohorts. A significant improvement from baseline of 16.0 (SD = 20.37) (*p* < 0.0196) points in mean EQ-5D VAS score was observed in the Reditux™ cohort as compared to a change by 8.3 (26.67) in the Ristova^®^ cohort after 2 years. BCL-2 expression and VAS were independent favorable prognostic factors for PFS.

## Data availability statement

The original contributions presented in the study are included in the article/**Supplementary Material**. Further inquiries can be directed to the corresponding author.

## Ethics statement

The studies involving humans were approved by 1.Tata Medical Centre, Kolkata 2. Rajiv Gandhi Cancer Institute and Research Centre Delhi 3. Jehangir Clinical Development Centre Pvt. Ltd. Pune, 4. Manipal Hospital Bangalore, 5. Nightingale Ethics Committe Kolkata. The studies were conducted in accordance with the local legislation and institutional requirements. The participants provided their written informed consent to participate in this study.

## Author contributions

The authors meet criteria for authorship as recommended by the International Committee of Medical Journal Editors (ICMJE). Each author in the study contributed with oversight and leadership responsibility for the research activity planning and execution. The authors received no direct compensation related to the development of the manuscript. RN, GB, NA, MS, PM and SN were the investigators among the competitive 29 investigating sites who were selected based on the recruitment. CL has worked as the external consultant to assist in analysing the data and in critical review of the manuscript. PR provided the statistical support and involved in data analysis and interpretation. SK and NM conceptualized the study and provided timely guidance in study conduct and data interpretation. All authors contributed to the article and approved the submitted version.

## References

[1] World Cancer Report 2020. International Agency for Research on Cancer (2020) (Accessed October 31, 2022).

[2] Nair R Arora N Mallath MK. Epidemiology of non-Hodgkin's lymphoma in India. Oncology (2016) 91(Suppl. 1):18–25. doi: 10.1159/000447577 27462703

[3] Salles G Barrett M Foà R Maurer J O’Brien S Valente N. Rituximab in B-cell hematologic Malignancies: a review of 20 years of clinical experience. Adv Ther (2017) 34:2232–73. doi: 10.1007/s12325-017-0612-x PMC5656728 28983798

[4] Doval D Bhurani DC Nair R Gujral S Malhotra P Ramanan G. Indian Council of Medical Research Consensus document for the management of non-Hodgkin’s lymphoma (high grade). Indian J Med Paediatric Oncol (2017) 38(01):51–8. doi: 10.4103/0971-5851.203500 PMC5398107 28469337

[5] Coiffier B Thieblemont C Van Den Neste E Lepeu G Plantier I Castaigne S. Long-term outcome of patients in the LNH-98.5 trial, the first randomized study comparing rituximab-CHOP to standard CHOP chemotherapy in DLBCL patients: a study by the Groupe d'Etudes des Lymphomes de l'Adulte. Blood J Am Soc Hematol (2010) 116(12):2040–5. doi: 10.1182/blood-2010-03-276246 PMC2951853 20548096

[6] Nair R Radhakrishnan VS Mallath MK. Rituximab biosimilars for B-cell lymphomas: a decade of real-world experience from India. Lancet Haematol (2021) 8(8):e548–9. doi: 10.1016/S2352-3026(21)00212-X 34329574

[7] Nava-Parada P Shelbaya A Nabhan C. Rituximab biosimilars in hematologic Malignancies: the need for a real-world approach. Future Oncol (2020) 16(26):2017–27. doi: 10.2217/fon-2020-0131 32598173

[8] Pierpont TM Limper CB Richards KL. Past, present, and future of rituximab—the world’s first oncology monoclonal antibody therapy. Front Oncol (2018) 8:163. doi: 10.3389/fonc.2018.00163 29915719 PMC5994406

[9] Qureshi ZP Magwood JS Singh S Bennett CL. Rituximab and biosimilars–equivalence and reciprocity. Biosimilars (2013) 2013(3):19. doi: 10.2147/BS.S20681 24829884 PMC4017581

[10] Viswabandya A Shah S Mukhopadhyay A Nagarkar RV Batra SS Lopez-Lazaro L. Randomized, double-blind, pharmacokinetic equivalence trial comparing DRL-rituximab with MabThera in patients with diffuse large B-cell lymphoma. J Global Oncol (2019) 5:1–3. doi: 10.1200/JGO.19.00248 PMC6939748 31809224

[11] Haridas VM Katta R Nalawade A Kharkar S Zhdan V Garmish O. Pharmacokinetic similarity and comparative pharmacodynamics, safety, efficacy, and immunogenicity of DRL_RI versus reference rituximab in biologics-naïve patients with moderate-to-severe rheumatoid arthritis: a double-blind, randomized, three-arm study. BioDrugs (2020) 34:183–96. doi: 10.1007/s40259-020-00406-1 PMC7113224 32052313

[12] Roy PS John S Karankal S Kannan S Pawaskar P Gawande J. Comparison of the efficacy and safety of Rituximab (Mabthera™) and its biosimilar (Reditux™) in diffuse large B-cell lymphoma patients treated with chemo-immunotherapy: A retrospective analysis. Indian J Med Paediatric Oncol (2013) 34(04):292–8. doi: 10.4103/0971-5851.125248 PMC3932598 24604960

[13] Bankar A Korula A Abraham A Viswabandya A George B Srivastava A. Comparison of the efficacy of innovator rituximab and its biosimilars in diffuse large B cell lymphoma patients: A retrospective analysis. Indian J Hematol Blood Transfusion (2020) 36:71–7. doi: 10.1007/s12288-019-01167-w PMC7042466 32174693

[14] Karthaus M Oskay-Özcelik G Wülfing P Hielscher C Guth D Zahn MO. Real-world evidence of NEPA, netupitant-palonosetron, in chemotherapy-induced nausea and vomiting prevention: effects on quality of life. Future Oncol (2020) 16(14):939–53. doi: 10.2217/fon-2020-0187 32298187

[15] Otremba B Borchardt J Kuske A Hollnagel-Schmitz M Losch FO. Real-world use and acceptance of rituximab biosimilars in non-Hodgkin lymphoma in an oncologist network in Germany. Future Oncol (2020) 16(15):1001–12. doi: 10.2217/fon-2020-0180 32286864

[16] World Medical Association. World Medical Association Declaration of Helsinki: ethical principles for medical research involving human subjects. JAMA (2013) 310(20):2191–4. doi: 10.1001/jama.2013.281053 24141714

[17] Campo E Swerdlow SH Harris NL Pileri S Stein H Jaffe ES. The 2008 WHO classification of lymphoid neoplasms and beyond: evolving concepts and practical applications. Blood J Am Soc Hematol (2011) 117(19):5019–32. doi: 10.1182/blood-2011-01-293050 PMC3109529 21300984

[18] Alizadeh AA Eisen MB Davis RE Ma C Lossos IS Rosenwald A. Distinct types of diffuse large B-cell lymphoma identified by gene expression profiling. Nature (2000) 403(6769):503–11. doi: 10.1038/35000501 10676951

[19] Cheson BD Pfistner B Juweid ME Gascoyne RD Specht L Horning SJ. Revised response criteria for Malignant lymphoma. J Clin Oncol (2007) 25(5):579–86. doi: 10.1200/JCO.2006.09.2403 17242396

[20] Heller GZ Manuguerra M Chow R. How to analyze the Visual Analogue Scale: Myths, truths and clinical relevance. Scandinavian J pain. (2016) 13(1):67–75. doi: 10.1016/j.sjpain.2016.06.012 28850536

[21] Cox DR. Regression models and life-tables. J R Stat Soc B: Stat Methodol (1972) 34(2):187–202.

[22] Gota V Karanam A Rath S Yadav A Tembhare P Subramanian P. Population pharmacokinetics of Reditux™, a biosimilar Rituximab, in diffuse large B-cell lymphoma. Cancer chemother Pharmacol (2016) 78:353–9. doi: 10.1007/s00280-016-3083-x 27329361

[23] Ganesan P Sagar TG Kannan K Radhakrishnan V Rajaraman S John A. Long-term outcome of diffuse large B-cell lymphoma: impact of biosimilar rituximab and radiation. Indian J Cancer (2017) 54(2):430–5. doi: 10.4103/ijc.IJC_241_17 29469072

[24] Prakash G Malhotra P Khadwal A Lad D Suri V Kumari S. Infusion related hypersensitivity reactions with bio-similar anti CD-20 monoclonal antibody rituximab in Indian patients: a retrospective study. Indian J Hematol Blood Transfusion (2018) 34:273–7. doi: 10.1007/s12288-017-0885-x PMC5884996 29622869

[25] Shun-Shin MJ Francis DP. Why even more clinical research studies may be false: effect of asymmetrical handling of clinically unexpected values. PloS One (2013) 8(6):e65323. doi: 10.1371/journal.pone.0065323 23825524 PMC3692492

[26] Blonde L Khunti K Harris SB Meizinger C Skolnik NS. Interpretation and impact of real-world clinical data for the practicing clinician. Adv Ther (2018) 35:1763–74. doi: 10.1007/s12325-018-0805-y PMC6223979 30357570

[27] Shelbaya A Kelton JM Thompson J Alvir JM Maculaitis MC Yang J. Real-world use and acceptance of biosimilar monoclonal antibodies of rituximab in oncology practice in the USA. Future Oncol (2021) 17(30):3941–50. doi: 10.2217/fon-2021-0618 34259584

[28] Franceschetti A Baskett A Baynton E Baldock D Karki C. PHP113- Comparative analysis of biosimilar rituximab usage in treating non-Hodgkin lymphoma and rheumatoid arthritis: results from a multi-country study in Europe. Value Health (2018) 21:S169.

[29] Horvat M Zadnik V Južnič Šetina T Boltežar L Pahole Goličnik J Novaković S. Diffuse large B-cell lymphoma: 10 years' real-world clinical experience with rituximab plus cyclophosphamide, doxorubicin, vincristine and prednisolone. Oncol Lett (2018) 15(3):3602–9. doi: 10.3892/ol.2018.7774 PMC5796369 29467881

[30] Salles G Seymour JF Offner F López-Guillermo A Belada D Xerri L. Rituximab maintenance for 2 years in patients with high tumour burden follicular lymphoma responding to rituximab plus chemotherapy (PRIMA): a phase 3, randomised controlled trial. Lancet (2011) 377(9759):42–51. doi: 10.1016/S0140-6736(10)62175-7 21176949

[31] Lehners N Krämer I Saadati M Benner A Ho AD Witzens-Harig M. Analysis of prognostic factors in patients with newly diagnosed diffuse large B-cell lymphoma and skeletal involvement. BMC Cancer (2017) 17(128):1–7. doi: 10.1186/s12885-017-3113-z 28193188 PMC5307829

[32] Iacoboni G Villacampa G Martinez-Cibrian N Bailén R Lopez Corral L Sanchez JM. Real-world evidence of tisagenlecleucel for the treatment of relapsed or refractory large B-cell lymphoma. Cancer Med (2021) 10(10):3214–23. doi: 10.1002/cam4.3881 PMC8124109 33932100

[33] Wu J Song Y Su L Xu L Chen T Zhao Z. Rituximab plus chemotherapy as first-line treatment in Chinese patients with diffuse large B-cell lymphoma in routine practice: a prospective, multicentre, non-interventional study. BMC Cancer (2016) 16(1):1–0. doi: 10.1186/s12885-016-2523-7 PMC4962436 27460571

[34] Kramer MH Hermans J Wijburg E Philippo K Geelen E Van Krieken JH. Clinical relevance of BCL2, BCL6, and MYC rearrangements in diffuse large B-cell lymphoma. Blood J Am Soc Hematol (1998) 92(9):3152–62. doi: 10.1182/blood.V92.9.3152 9787151

[35] Iqbal J Meyer PN Smith LM Johnson NA Vose JM Greiner TC. BCL2 predicts survival in germinal center B-cell–like diffuse large B-cell lymphoma treated with CHOP-like therapy and rituximab. Clin Cancer Res (2011) 17(24):7785–95. doi: 10.1158/1078-0432.CCR-11-0267 PMC7394278 21933893

[36] Lossos IS Czerwinski DK Alizadeh AA Wechser MA Tibshirani R Botstein D. Prediction of survival in diffuse large-B-cell lymphoma based on the expression of six genes. New Engl J Med (2004) 350(18):1828–37. doi: 10.1056/NEJMoa032520 15115829

[37] Hans CP Weisenburger DD Greiner TC Gascoyne RD Delabie J Ott G. Confirmation of the molecular classification of diffuse large B-cell lymphoma by immunohistochemistry using a tissue microarray. Blood (2004) 103(1):275–82. doi: 10.1182/blood-2003-05-1545 14504078

[38] Roche N Reddel H Martin R Brusselle G Papi A Thomas M. Quality standards for real-world research. Focus on observational database studies of comparative effectiveness. Ann Am Thorac Soc (2014) 11(Supplement 2):S99–104. doi: 10.1513/AnnalsATS.201309-300RM 24559028

[39] RITUXAN® Injection Genentech (997) (Accessed 02 March 2022). Published September 2019.

