# Supplementary material for: Real-world outcomes of diffuse large B-cell lymphoma in the biosimilar era
Source: Front Oncol. 2023 Oct 3;13:1248723. doi: 10.3389/fonc.2023.1248723 (PMC10580068; doi:10.3389/fonc.2023.1248723)
Supplement: Supplementary file 1 [file Table_1.docx]

## Supplementary data

**Title: Real-world outcomes of Diffuse Large B-Cell Lymphoma in the Biosimilars era**

**Authors:** Reena Nair^a^, Gull Mohammad Bhat^b^, Narendra Agrawal^c^, Manju Sengar^d^, Pankaj Malhotra^e^, Soniya Nityanand^f^, Chitra Lele^g^, Pramod Reddy*^h^, Suresh Kankanwadi^i^, Narendra Maharaj^h^.

**Affiliations:** ^a^ Department of Clinical Haematology, Tata Medical Centre, Kolkata, West Bengal, India.

^b^Department of Medical Oncology, Sher-I-Kashmir Institute of Medical Sciences, Srinagar, Jammu & Kashmir, India.

^c^Department of Hemato-Oncology & [Bone Marrow Transplant](https://www.rgcirc.org/specialties/bone-marrow-transplantation/), Rajiv Gandhi Cancer Institute and Research Center, Delhi, India.

^d^Adult hematolymphoid disease management group, Medical Oncology, Tata Memorial Hospital, Mumbai, Maharashtra, India.

^e^Department of Clinical Hematology & Medical Oncology, PGIMER, Chandigarh, India.

^f^Department of Hemotology, Sanjay Gandhi Post Graduate Institute of Medical Sciences, Lucknow, Uttar Pradesh, India.

^g^ActuReal Services and consulting Private limited, Pune, Maharashtra, India.

^h^Dr. Reddy’s Laboratories Ltd, Biologics, Bachupally, Hyderabad, India.

^i^Ex-Dr. Reddy’s Laboratories Ltd, Biologics, Bachupally, Hyderabad, India.

**^*^Corresponding Author**: Pramod Reddy,

Dr. Reddy’s Laboratories Ltd., Biologics

Survey No. 47, Bachupally Village,

Bachupally Mandal, Medchal Malkajgiri District,

Telangana, India - 500 090, INDIA

Email: [pramodkrp@drreddys.com](mailto:pramodkrp@drreddys.com)

Phone: +91 40-4464-4000

Fax: +91 40-2304-1418

**Supplementary Table 1**: **Summary of chemotherapy treatments at baseline**

|  | Reditux (N=1250) | Ristova (N=115) |
| --- | --- | --- |
| Number of subjects excluded from analysis* | 151 (312.1) | 3 (2.6) |
|  | | |
| Number of subjects used in analysis* (N1) | 1099 (87.9) | 112 (97.4) |
|  | | |
| CHOP-R | 684 (62.2) | 64 (57.1) |
| CHOP-Like therapies with R | 50 (4.6) | 2 (1.8) |
| Rituximab monotherapy | 15 (1.4) | 2 (1.8) |
| Others | 350 (31.8) | 44 (39.3) |

*: Percentages are based on number of subjects in each treatment group (N) at baseline as denominator.
Percentages for the Classes are based on total number of subjects used in analysis (N1) at baseline as denominator.

**Classification of Chemotherapy regimen**; based on as mentioned below:
**CHOP-R Standard therapies** (include CHOP and its variants (like daunorubicin instead of doxorubicin, CHOEP, changes in glucocorticoids- like Prednisolone, Dexamethasone, etc).
**CHOP-like therapies including R**, (include anthracycline containing chemotherapies- Mitoxantrone, doxorubixcin, daunorubicin, epirubicin and idarubicin).
**Rituximab monotherapy** (includes no other chemotherapies).
**Others-** chemotherapies other than any of the above include immune-modulators and targeted agents like lenalidomide and ibrutinib

**Supplementary Table 2:**  **Summary of demographics by progression of disease at 2 years among Reditux^TM^ and Ristova^®^ treatment cohorts**

|  | **Reditux^TM^** | | | | **Ristova^®^** | | | | | | | | |
| --- | --- | --- | --- | --- | --- | --- | --- | --- | --- | --- | --- | --- | --- |
| Characteristics   Statistics | Disease not progressed   (N=791) | Disease progressed   (N=355) | Mean Difference/   Proportion (95% CI) | p-value | Disease not progressed   (N=79) | | | Disease progressed   (N=31) | | Mean Difference (95% CI) | | | p-value |
| Age (in years) | | | | |  | | | | | | | | |
| n | 791 | 355 |  |  | 79 | | | 31 | |  | | |  |
| Mean (S.Dev) | 51.7 (14.58) | 55.8 (15.33) | -4.15 (-6.00, -2.29) | <0.0001* | 57.5 (13.87) | | | 60.4 (15.31) | | -2.90 (-8.90, 3.10) | | | 0.3401* |
| Median | 53.3 | 59.2 |  |  | 58.0 | | | 64.3 | |  | | |  |
| Range | (18.1, 88.2) | (19.0, 88.5) |  |  | (22.9, 80.9) | | | (20.0, 86.0) | |  | | |  |
|  |  |  |  |  |  | | |  | |  | | |  |
| BMI (kg/m^2^) |  |  |  |  |  | | |  | |  | | |  |
| n | 711 | 325 |  |  | 78 | | | 28 | |  | | |  |
| Mean (S.Dev) | 23.9 (4.50) | 23.6 (4.96) | 0.32 (-0.31, 0.95) | 0.3224* | 26.3 (5.84) | | | 24.6 (3.39) | | 1.71 (-0.12, 3.54) | | | 0.0671* |
| Median | 23.6 | 23.2 |  |  | 25.4 | | | 25.0 | |  | | |  |
| Range | (13.2, 47.9) | (12.5, 46.8) |  |  | (16.2, 53.0) | | | (18.1, 30.1) | |  | | |  |
| Missing | 80 (10.1) | 30(8.5) |  |  | 1 (1.3) | 3 (9.7) | | | | |  |  | |
|  |  |  |  |  |  | | |  | |  | | |  |
| Height (in cm) |  |  |  |  |  | | |  | |  | | |  |
| n | 725 | 326 |  |  | 78 | | | 28 | |  | | |  |
| Mean (S.Dev) | 161.6 (9.10) | 161.8 (8.75) | -0.17 (-1.35, 1.01) | 0.7775* | 162.3 (10.39) | | | 163.3 (10.26) | | -0.95 (-5.48, 3.57) | | | 0.6772* |
| Median | 162.0 | 162.0 |  |  | 164.0 | | | 165.5 | |  | | |  |
| Range | 115.0,191.0 | 140.0, 187.0 |  |  | (132.0, 184.0) | | | | | | | | |
| Missing | 66 (8.3) | 29 (8.2) |  |  | 1 (1.3) | | | 3 (9.7) | |  | | |  |
|  |  |  |  |  |  | | |  | |  | | |  |
| Weight (in Kg) |  |  |  |  |  | | |  | |  | | |  |
| n | 726 | 329 |  |  | 78 | | | 29 | |  | | |  |
| Mean (S.Dev) | 62.4 (13.14) | 61.7 (13.73) | 0.70 (-1.04, 2.44) | 0.4277* | 69.6 (18.18) | | | 65.6 (10.76) | | 4.00 (-1.70, 9.71) | | | 0.1665* |
| Median | 61.0 | 60.4 |  |  | 65.5 | | 63.4 | |  | | | |  |
| Range | 31.0, 164.0 | 34.0, 114.0 |  |  | (38.0, 160.5) | | | (50.0, 87.2) | |  | | |  |
| Missing | 65 (8.2) | 26 (7.3) |  |  | 1(1.3) | | | 2(6.5) | |  | | |  |
|  |  |  |  |  |  | | |  | |  | | |  |
| Gender, n (%) | | | |  |  | | | | | | | |  |
| Female | 265 (33.5) | 127 (35.8) | -2.27 (-8.25,3.70) | 0.4533** | 32 (40.5) | | | 11 (35.5) | | 5.02 (-15.00,25.04) | | | 0.6272** |
| Male | 526 (66.5) | 228 (64.2) |  |  | 47 (59.5) | | | 20 (64.5) | |  | | |  |
|  |  |  |  |  |  | | |  | |  | | |  |
| Education Level Completed, n (%) | | | |  |  | | | | | | | |  |
| Primary education | 230 (29.6) | 100 (28.6) | 1.07 (-4.65,6.79) | 0.7156** | 7 (10.3) | | | 7 (25.9) | | -15.63 (-33.67,2.41) | | | 0.0525** |
| Secondary education | 208 (26.8) | 114 (32.6) |  |  | 17 (25.0) | | | 6 (22.2) | |  | | |  |
| Higher education | 312 (40.2) | 124 (35.4) |  |  | 44 (64.7) | | | 14 (51.9) | |  | | |  |
| No education level completed | 26 (3.4) | 12 (3.4) |  |  |  | | |  | |  | | |  |
| Missing | 15 (1.9) | 5 (1.4) |  |  | 11 (13.9) | | 4 (12.9) | |  | | | |  |
|  |  |  |  |  |  | | |  | |  | | |  |
| Smoking Status, n (%) |  |  |  |  |  | | |  | |  | | |  |
| Current | 44 (5.6) | 16 (4.5) | 1.06 (-1.63,3.74) | 0.4134** | 1 (1.3) | | | 1 (3.2) | | -1.96 (-8.65,4.73) | | | 0.0010** |
| Past | 126 (15.9) | 68 (19.2) |  |  | 5 (6.3) | | | 9 (29.0) | |  | | |  |
| Never | 621 (78.5) | 271 (76.3) |  |  | 73 (92.4) | | | 21 (67.7) | |  | | |  |
| Type of Hospital, n (%) |  |  |  |  |  | | |  | |  | | |  |
| Private | 258 (32.6) | 109 (30.7) | 1.91 (-3.89,7.72) | 0.5211** | 53 (67.1) | | | 20 (64.5) | | 2.57 (-17.20,22.35) | | | 0.7972** |
| Academic | 306 (38.7) | 116 (32.7) |  |  | 25 (31.6) | | | 9 (29.0) | |  | | |  |
| Government | 227 (28.7) | 130 (36.6) |  |  | 1 (1.3) | | | 2 (6.5) | |  | | |  |
|  |  |  |  |  |  | | |  | |  | | |  |

N: Number of subjects for which Progression of disease (Yes/No) has been captured.
n: Number of subject with specific characteristics.
Percentages are calculated using number of subjects with non-missing data for Progression of disease (Yes/No) as a denominator.
Percentages for missing category are calculated using number of subjects (N) with data for Progression of disease (Yes/No) as a denominator.
#: Percentages are calculated using number of subject with diagnosis data as YES and Progression of disease (Yes/No) as a denominator.
*: p-values are obtained using two sample t-test.
**: p-values are obtained using chi-square test or fisher exact test (for small sample size).
^: Patients may have multiple Genomic Data-Subtypes and hence p-value and CI's are not displayed.

**Supplementary Table 3**: **Summary of baseline clinical characteristics by progression of disease at 2 years among Reditux^TM^ and Ristova^®^ treatment cohorts**

|  | **Reditux^TM^** | | | | **Ristova^®^** | | | |
| --- | --- | --- | --- | --- | --- | --- | --- | --- |
| **Demographics/ Characteristics /  Statistics** | **Disease not progressed   (N=791)** | **Disease progressed   (N=355)** | **Mean Difference/Proportion**  **(95% CI)** | **p-value** | **Disease not progressed   (N=79)** | **Disease progressed   (N=31)** | **Mean Difference/   Proportion (95% CI)** | **p-value** |
| Duration of Disease (month) | | | |  | | | | |
| n | 752 | 336 |  |  | 77 | 29 |  |  |
| Mean (SD) | 1.2 (2.87) | 1.3 (2.13) | -0.02 (-0.33,0.28) | 0.8796* | 0.9 (0.51) | 1.0 (0.63) | -0.14 (-0.37,0.10) | 0.2570* |
| Median | 0.9 | 0.9 |  |  | 0.8 | 0.9 |  |  |
| Range | (0.0, 68.0) | (0.0, 31.0) |  |  | (0.2, 2.4) | (0.1, 3.0) |  |  |
| Missing | 39 (4.9) | 19 (5.4) |  |  | 2 (2.5) | 2 (6.5) |  |  |
| Age at Diagnosis | | | |  |  | | | |
| n | 752 | 336 |  |  | 77 | 29 |  |  |
| Mean (SD) | 52.0 (14.60) | 56.2 (15.21) | -4.22 (-6.12,-2.31) | <0.0001* | 57.2 (13.91) | 59.6 (15.47) | -2.31 (-8.51,3.88) | 0.4606* |
| Median | 53.6 | 59.9 |  |  | 57.8 | 61.8 |  |  |
| Range | (18.0, 88.1) | (19.1, 88.4) |  |  | (22.9, 80.9) | (20.0, 86.7) |  |  |
| Missing | 39 (4.9) | 19 (5.4) |  |  | 2 (2.5) | 2 (6.5) |  |  |
| DLBCL Staging |  |  |  |  |  |  |  |  |
| Ann Arbor Stage |  |  |  | 0.0008** |  |  |  | 0.3989** |
| Localized  (Stage I or II) | 285 (43.6) | 88 (31.9) |  |  | 27 (45.0) | 8 (34.8) |  |  |
| Advanced  (Stage III or IV) | 368 (56.4) | 188 (68.1) | -11.76 (-18.45,-5.08) |  | 33 (55.0) | 15 (65.2) | -10.22 (-33.40,12.96) |  |
| Missing | 138 (17.4) | 79 (22.3) |  |  | 19 (24.1) | 8 (25.8) |  |  |
| ECOG-PS: (0-5, 5=Death), n (%) | | | | 0.0008** |  |  |  | 0.8605** |
| Better performance (< 2) | 481 (73.7) | 173 (62.7) |  |  | 48 (80.0) | 18 (78.3) |  |  |
| Worst performance (2 to 4) | 172 (26.3) | 103 (37.3) | -10.98 (-17.61,-4.35) |  | 12 (20.0) | 5 (21.7) | -1.74 (-21.40,17.92) |  |
| Missing | 138 (17.4) | 79 (22.3) |  |  | 19 (24.1) | 8 (25.8) |  |  |
|  |  |  |  |  |  |  |  |  |
| IPI Age, n (%) | | | | <0.0001** |  |  |  | 0.2467** |
| <=60 | 439 (67.4) | 140 (50.9) |  |  | 32 (53.3) | 9 (39.1) |  |  |
| >60 | 212 (32.6) | 135 (49.1) | -16.53 (-23.44,-9.61) |  | 28 (46.7) | 14 (60.9) | -14.20 (-37.81,9.40) |  |
| Missing | 140 (17.7) | 80 (22.5) |  |  | 19 (24.1) | 8 (25.8) |  |  |
| Elevated Serum LDH, n (%) | | | | 0.0024** |  |  |  | 0.9708** |
| No | 318 (48.7) | 104 (37.8) |  |  | 33 (55.0) | 12 (54.5) |  |  |
| Yes | 335 (51.3) | 171 (62.2) | -10.88 (-17.78,-3.98) |  | 27 (45.0) | 10 (45.5) | -0.45 (-24.77,23.86) |  |
| Missing | 138 (17.4) | 80 (22.5) |  |  | 19 (24.1) | 9 (29.0) |  |  |
| More than 1 Extranodal  site, n (%) | | | |  |  |  |  | 0.4032** |
| No | 423 (64.8) | 151 (54.7) |  | 0.0039** | 47 (78.3) | 16 (69.6) |  |  |
| Yes | 230 (35.2) | 125 (45.3) | -10.07 (-16.99,-3.15) |  | 13 (21.7) | 7 (30.4) | -8.77 (-30.27,12.73 |  |
| Missing | 138 (17.4) | 79 (22.3) |  |  | 19 (24.1) | 8 (25.8) |  |  |
| IPI, n (%) |  |  |  | 0.0008** |  |  |  | 0.2721** |
| Good risk group  (IPI score, 0-2) | 518 (68.1) | 194 (57.6) |  |  | 54 (71.1) | 18 (60.0) |  |  |
| Poor risk group  (IPI score, 3-5) | 243 (31.9) | 143 (42.4) | -10.50 (-16.73,-4.27) |  | 22 (28.9) | 12 (40.0) | -11.05 (-31.33,9.23) |  |
| Missing | 30 (3.8) | 18 (5.1) |  |  | 3 (3.8) | 1 (3.2) |  |  |
| Charlson Major Comorbidity Index, n (%) | | | | 0.0779** |  | | | 0.4231** |
| Low CCI ( 0 or 1) | 405 (51.3) | 162 (45.6) |  |  | 47 (59.5) | 21 (67.7) |  |  |
| Moderate to High CCI (2 or more) | 385 (48.7) | 193 (54.4) | -5.63 (-11.88,0.61) |  | 32 (40.5) | 10 (32.3) | 8.25 (-11.45,27.95) |  |
| Missing | 1 (0.1) | 0 |  |  | 0 | 0 |  |  |
|  |  |  |  |  |  |  |  |  |
| Visual Analogue Scale (EQ5D) | | | | | | | | |
| n | 782 | 353 |  |  | 79 | 31 |  |  |
| Mean(S.Dev) | 64.6 (18.73) | 60.8 (19.19) | 3.76 (1.38,6.13) | 0.0020* | 74.0 (19.14) | 64.0 (24.57) | 9.96 (1.23,18.70) | 0.0257* |
| Median | 65.0 | 60.0 |  |  | 80.0 | 70.0 |  |  |
| Range | (0.0, 100.0) | (10.0, 100.0) |  |  | (20.0, 100.0) | (4.7, 100.0) |  |  |
| Missing | 9 (1.1) | 2 (0.6) |  |  | 0 | 0 |  |  |
| DLBCL Diagnosis Classification n (%)# | | | | 0.1529** |  | | | 0.0199** |
| Activated B-cell (ABC)-DLBCL | 187 (28.2) | 92 (34.6) | -6.38 (-13.05,0.28) |  | 15 (21.1) | 10 (41.7) | -20.54 (-42.43,1.35) |  |
| Germinal center B-cell (GCB)-DLBCL | 221 (33.3) | 84 (31.6) | 1.75 (-4.88,8.39) |  | 27 (38.0) | 4 (16.7) | 21.36 (2.66,40.06) |  |
| Intravascular large B-cell lymphoma | 0 | 1 (0.4) | -0.38 (-1.11,0.36) |  | 0 | 0 |  |  |
| Primary DLBCL of the central nervous system (CNS) | 12 (1.8) | 4 (1.5) | 0.31 (-1.47,2.09) |  | 0 | 1 (4.2) | -4.17 (-12.16,3.83) |  |
| Primary cutaneous DLBCL- leg type | 6 (0.9) | 5 (1.9) | -0.97 (-2.76,0.81) |  | 0 | 0 |  |  |
| Primary mediastinal lymphoma | 4 (0.6) | 0 | 0.60 (0.01,1.19) |  | 1 (1.4) | 0 | 1.41 (-1.33,4.15) |  |
| T-cell/histiocyte rich large B-cell lymphoma | 57 (8.6) | 21 (7.9) | 0.70 (-3.18,4.58) |  | 8 (11.3) | 3 (12.5) | -1.23 (-16.37,13.91) |  |
| IHC Data-Subtypes, n (%)^ | | | | | | | | |
| **BCL2 (IHC)** | | | | | | | | |
| Yes | 322 (45.8) | 152 (48.7) |  |  | 38 (52.1) | 14 (50.0) |  |  |
| No | 153 (21.8) | 46 (14.7) |  |  | 17 (23.3) | 4 (14.3) |  |  |
| Not Available | 228 (32.4) | 114 (36.5) |  |  | 18 (24.7) | 10 (35.7) |  |  |
| Missing | 88 (11.1) | 43 (12.1) |  |  | 6 (7.6) | 3 (9.7) |  |  |
| **BCL6 (IHC)** | | | | | | | | |
| Yes | 385 (54.8) | 151 (48.4) |  |  | 39 (53.4) | 11 (39.3) |  |  |
| No | 105 (14.9) | 52 (16.7) |  |  | 12 (16.4) | 6 (21.4) |  |  |
| Not Available | 213 (30.3) | 109 (34.9) |  |  | 22 (30.1) | 11 (39.3) |  |  |
| Missing | 88 (11.1) | 43 (12.1) |  |  | 6 (7.6) | 3 (9.7) |  |  |
| **CD10 (IHC)** | | | | | | | | |
| Yes | 257 (36.6) | 104 (33.3) |  |  | 33 (45.2) | 10 (35.7) |  |  |
| No | 301 (42.8) | 132 (42.3) |  |  | 23 (31.5) | 8 (28.6) |  |  |
| Not Available | 145 (20.6) | 76 (24.4) |  |  | 17 (23.3) | 10 (35.7) |  |  |
| Missing | 88 (11.1) | 43 (12.1) |  |  | 6 (7.6) | 3 (9.7) |  |  |
| **MUM1/IRF4 (IHC)** | | | | | | | | |
| Yes | 331 (47.1) | 148 (47.4) |  |  | 35 (47.9) | 16 (57.1) |  |  |
| No | 147 (20.9) | 61 (19.6) |  |  | 13 (17.8) | 2 (7.1) |  |  |
| Not Available | 225 (32.0) | 103 (33.0) |  |  | 25 (34.2) | 10 (35.7) |  |  |
| Missing | 88 (11.1) | 43 (12.1) |  |  | 6 (7.6) | 3 (9.7) |  |  |
|  | | | | | | | | |
| **MYC** | | | | | | | | |
| Yes | 18 (2.6) | 12 (3.8) |  |  | 7 (9.6) | 2 (7.1) |  |  |
| No | 76 (10.8) | 29 (9.3) |  |  | 12 (16.4) | 4 (14.3) |  |  |
| Not Available | 609 (86.6) | 271 (86.9) |  |  | 54 (74.0) | 22 (78.6) |  |  |
| Missing | 88 (11.1) | 43 (12.1) |  |  | 6 (7.6) | 3 (9.7) |  |  |
|  | | | | | | | | |
|  |  |  |  |  |  |  |  |  |
|  |  |  |  |  |  |  |  |  |

N: Number of subjects for which Progression of disease(Yes/No) has been captured.
n: Number of subject with specific characteristics.
Percentages are calculated using number of subjects with non-missing data for Progression of disease (Yes/No) as a denominator.
Percentages for missing category are calculated using number of subjects (N) with data for Progression of disease (Yes/No) as a denominator.
#:Percentages are calculated using number of subject with diagnosis data as YES and Progression of disease(Yes/No) as a denominator.
*: p-values are obtained using two sample t-test.
**: p-values are obtained using chi-square test or fisher exact test (for small sample size).
^: Patients may have multiple IHC Data-Subtypes and hence p-value and CI's are not displayed.

**Supplementary Table 4: Summary of Treatment Related Adverse Events by Maximum Severity Grade-DLBCL**

| System Organ Class  Preferred Term, n (%) | Severity Grade | Reditux  (N=1250) | Ristova  (N=115) | Total  (N=1365) |
| --- | --- | --- | --- | --- |
| Subjects with At Least one AE | Total | 47 (3.8) | 4 (3.5) | 51 (3.7) |
|  | 1 | 15 (1.2) | 2 (1.7) | 17 (1.2) |
|  | 2 | 12 (1.0) | 0 | 12 (0.9) |
|  | 3 | 13 (1.0) | 2 (1.7) | 15 (1.1) |
|  | 4 | 4 (0.3) | 0 | 4 (0.3) |
|  | 5 | 9 (0.7) | 1 (0.9) | 10 (0.7) |
|  |  |  |  |  |
| Blood And Lymphatic System Disorders | Total | 18 (1.4) | 2 (1.7) | 20 (1.5) |
|  | 1 | 5 (0.4) | 2 (1.7) | 7 (0.5) |
|  | 2 | 2 (0.2) | 0 | 2 (0.1) |
|  | 3 | 7 (0.6) | 0 | 7 (0.5) |
|  | 4 | 3 (0.2) | 0 | 3 (0.2) |
|  | 5 | 1 (0.1) | 0 | 1 (0.1) |
|  |  |  |  |  |
| Febrile Neutropenia | Total | 12 (1.0) | 2 (1.7) | 14 (1.0) |
|  | 1 | 3 (0.2) | 2 (1.7) | 5 (0.4) |
|  | 2 | 2 (0.2) | 0 | 2 (0.1) |
|  | 3 | 5 (0.4) | 0 | 5 (0.4) |
|  | 4 | 2 (0.2) | 0 | 2 (0.1) |
|  | 5 | 0 | 0 | 0 |
|  |  |  |  |  |
| Neutropenia | Total | 6 (0.5) | 0 | 6 (0.4) |
|  | 1 | 1 (0.1) | 0 | 1 (0.1) |
|  | 2 | 1 (0.1) | 0 | 1 (0.1) |
|  | 3 | 3 (0.2) | 0 | 3 (0.2) |
|  | 4 | 1 (0.1) | 0 | 1 (0.1) |
|  | 5 | 0 | 0 | 0 |
|  |  |  |  |  |
| Anaemia | Total | 2 (0.2) | 0 | 2 (0.1) |
|  | 1 | 1 (0.1) | 0 | 1 (0.1) |
|  | 2 | 0 | 0 | 0 |
|  | 3 | 0 | 0 | 0 |
|  | 4 | 0 | 0 | 0 |
|  | 5 | 1 (0.1) | 0 | 1 (0.1) |
|  |  |  |  |  |
| Cardiac Disorders | Total | 4 (0.3) | 1 (0.9) | 5 (0.4) |
|  | 1 | 2 (0.2) | 0 | 2 (0.1) |
|  | 2 | 0 | 0 | 0 |
|  | 3 | 1 (0.1) | 0 | 1 (0.1) |
|  | 4 | 1 (0.1) | 0 | 1 (0.1) |
|  | 5 | 0 | 1 (0.9) | 1 (0.1) |
|  |  |  |  |  |
| Tachycardia | Total | 3 (0.2) | 0 | 3 (0.2) |
|  | 1 | 2 (0.2) | 0 | 2 (0.1) |
|  | 2 | 0 | 0 | 0 |
|  | 3 | 0 | 0 | 0 |
|  | 4 | 1 (0.1) | 0 | 1 (0.1) |
|  | 5 | 0 | 0 | 0 |
|  |  |  |  |  |
| Left Ventricular Dysfunction | Total | 1 (0.1) | 0 | 1 (0.1) |
|  | 1 | 0 | 0 | 0 |
|  | 2 | 0 | 0 | 0 |
|  | 3 | 1 (0.1) | 0 | 1 (0.1) |
|  | 4 | 0 | 0 | 0 |
|  | 5 | 0 | 0 | 0 |
|  |  |  |  |  |
| Myocardial Ischaemia | Total | 0 | 1 (0.9) | 1 (0.1) |
|  | 1 | 0 | 0 | 0 |
|  | 2 | 0 | 0 | 0 |
|  | 3 | 0 | 0 | 0 |
|  | 4 | 0 | 0 | 0 |
|  | 5 | 0 | 1 (0.9) | 1 (0.1) |
|  |  |  |  |  |
| Ear And Labyrinth Disorders | Total | 2 (0.2) | 0 | 2 (0.1) |
|  | 1 | 2 (0.2) | 0 | 2 (0.1) |
|  | 2 | 0 | 0 | 0 |
|  | 3 | 0 | 0 | 0 |
|  | 4 | 0 | 0 | 0 |
|  | 5 | 0 | 0 | 0 |
|  |  |  |  |  |
| Hypoacusis | Total | 2 (0.2) | 0 | 2 (0.1) |
|  | 1 | 2 (0.2) | 0 | 2 (0.1) |
|  | 2 | 0 | 0 | 0 |
|  | 3 | 0 | 0 | 0 |
|  | 4 | 0 | 0 | 0 |
|  | 5 | 0 | 0 | 0 |
|  |  |  |  |  |
| Eye Disorders | Total | 1 (0.1) | 0 | 1 (0.1) |
|  | 1 | 0 | 0 | 0 |
|  | 2 | 1 (0.1) | 0 | 1 (0.1) |
|  | 3 | 0 | 0 | 0 |
|  | 4 | 0 | 0 | 0 |
|  | 5 | 0 | 0 | 0 |
|  |  |  |  |  |
| Vision Blurred | Total | 1 (0.1) | 0 | 1 (0.1) |
|  | 1 | 0 | 0 | 0 |
|  | 2 | 1 (0.1) | 0 | 1 (0.1) |
|  | 3 | 0 | 0 | 0 |
|  | 4 | 0 | 0 | 0 |
|  | 5 | 0 | 0 | 0 |
|  |  |  |  |  |
| Faecal Volume Increased | Total | 1 (0.1) | 0 | 1 (0.1) |
|  | 1 | 1 (0.1) | 0 | 1 (0.1) |
|  | 2 | 0 | 0 | 0 |
|  | 3 | 0 | 0 | 0 |
|  | 4 | 0 | 0 | 0 |
|  | 5 | 0 | 0 | 0 |
|  |  |  |  |  |
| Faecal Volume Increased | Total | 1 (0.1) | 0 | 1 (0.1) |
|  | 1 | 1 (0.1) | 0 | 1 (0.1) |
|  | 2 | 0 | 0 | 0 |
|  | 3 | 0 | 0 | 0 |
|  | 4 | 0 | 0 | 0 |
|  | 5 | 0 | 0 | 0 |
|  |  |  |  |  |
| Gastrointestinal Disorders | Total | 11 (0.9) | 1 (0.9) | 12 (0.9) |
|  | 1 | 6 (0.5) | 0 | 6 (0.4) |
|  | 2 | 5 (0.4) | 0 | 5 (0.4) |
|  | 3 | 0 | 1 (0.9) | 1 (0.1) |
|  | 4 | 0 | 0 | 0 |
|  | 5 | 0 | 0 | 0 |
|  |  |  |  |  |
| Vomiting | Total | 5 (0.4) | 1 (0.9) | 6 (0.4) |
|  | 1 | 2 (0.2) | 1 (0.9) | 3 (0.2) |
|  | 2 | 3 (0.2) | 0 | 3 (0.2) |
|  | 3 | 0 | 0 | 0 |
|  | 4 | 0 | 0 | 0 |
|  | 5 | 0 | 0 | 0 |
|  |  |  |  |  |
| Constipation | Total | 4 (0.3) | 1 (0.9) | 5 (0.4) |
|  | 1 | 2 (0.2) | 0 | 2 (0.1) |
|  | 2 | 2 (0.2) | 0 | 2 (0.1) |
|  | 3 | 0 | 1 (0.9) | 1 (0.1) |
|  | 4 | 0 | 0 | 0 |
|  | 5 | 0 | 0 | 0 |
|  |  |  |  |  |
| Diarrhoea | Total | 3 (0.2) | 0 | 3 (0.2) |
|  | 1 | 2 (0.2) | 0 | 2 (0.1) |
|  | 2 | 1 (0.1) | 0 | 1 (0.1) |
|  | 3 | 0 | 0 | 0 |
|  | 4 | 0 | 0 | 0 |
|  | 5 | 0 | 0 | 0 |
|  |  |  |  |  |
| Abdominal Pain | Total | 1 (0.1) | 1 (0.9) | 2 (0.1) |
|  | 1 | 0 | 0 | 0 |
|  | 2 | 1 (0.1) | 1 (0.9) | 2 (0.1) |
|  | 3 | 0 | 0 | 0 |
|  | 4 | 0 | 0 | 0 |
|  | 5 | 0 | 0 | 0 |
|  |  |  |  |  |
| Nausea | Total | 2 (0.2) | 0 | 2 (0.1) |
|  | 1 | 2 (0.2) | 0 | 2 (0.1) |
|  | 2 | 0 | 0 | 0 |
|  | 3 | 0 | 0 | 0 |
|  | 4 | 0 | 0 | 0 |
|  | 5 | 0 | 0 | 0 |
|  |  |  |  |  |
| Lip Swelling | Total | 1 (0.1) | 0 | 1 (0.1) |
|  | 1 | 0 | 0 | 0 |
|  | 2 | 1 (0.1) | 0 | 1 (0.1) |
|  | 3 | 0 | 0 | 0 |
|  | 4 | 0 | 0 | 0 |
|  | 5 | 0 | 0 | 0 |
|  |  |  |  |  |
| Oral Disorder | Total | 1 (0.1) | 0 | 1 (0.1) |
|  | 1 | 0 | 0 | 0 |
|  | 2 | 1 (0.1) | 0 | 1 (0.1) |
|  | 3 | 0 | 0 | 0 |
|  | 4 | 0 | 0 | 0 |
|  | 5 | 0 | 0 | 0 |
|  |  |  |  |  |
| General Disorders And Administration Site Conditions | Total | 20 (1.6) | 1 (0.9) | 21 (1.5) |
|  | 1 | 4 (0.3) | 0 | 4 (0.3) |
|  | 2 | 4 (0.3) | 0 | 4 (0.3) |
|  | 3 | 4 (0.3) | 0 | 4 (0.3) |
|  | 4 | 0 | 0 | 0 |
|  | 5 | 8 (0.6) | 1 (0.9) | 9 (0.7) |
|  |  |  |  |  |
| Pyrexia | Total | 8 (0.6) | 0 | 8 (0.6) |
|  | 1 | 3 (0.2) | 0 | 3 (0.2) |
|  | 2 | 3 (0.2) | 0 | 3 (0.2) |
|  | 3 | 2 (0.2) | 0 | 2 (0.1) |
|  | 4 | 0 | 0 | 0 |
|  | 5 | 0 | 0 | 0 |
|  |  |  |  |  |
| Death | Total | 5 (0.4) | 0 | 5 (0.4) |
|  | 1 | 0 | 0 | 0 |
|  | 2 | 0 | 0 | 0 |
|  | 3 | 0 | 0 | 0 |
|  | 4 | 0 | 0 | 0 |
|  | 5 | 5 (0.4) | 0 | 5 (0.4) |
|  |  |  |  |  |
| Mucosal Inflammation | Total | 4 (0.3) | 0 | 4 (0.3) |
|  | 1 | 1 (0.1) | 0 | 1 (0.1) |
|  | 2 | 1 (0.1) | 0 | 1 (0.1) |
|  | 3 | 2 (0.2) | 0 | 2 (0.1) |
|  | 4 | 0 | 0 | 0 |
|  | 5 | 0 | 0 | 0 |
|  |  |  |  |  |
| Asthenia | Total | 3 (0.2) | 0 | 3 (0.2) |
|  | 1 | 2 (0.2) | 0 | 2 (0.1) |
|  | 2 | 1 (0.1) | 0 | 1 (0.1) |
|  | 3 | 0 | 0 | 0 |
|  | 4 | 0 | 0 | 0 |
|  | 5 | 0 | 0 | 0 |
|  |  |  |  |  |
| Pain | Total | 3 (0.2) | 0 | 3 (0.2) |
|  | 1 | 1 (0.1) | 0 | 1 (0.1) |
|  | 2 | 2 (0.2) | 0 | 2 (0.1) |
|  | 3 | 0 | 0 | 0 |
|  | 4 | 0 | 0 | 0 |
|  | 5 | 0 | 0 | 0 |
|  |  |  |  |  |
| Unevaluable Event | Total | 2 (0.2) | 0 | 2 (0.1) |
|  | 1 | 0 | 0 | 0 |
|  | 2 | 0 | 0 | 0 |
|  | 3 | 0 | 0 | 0 |
|  | 4 | 0 | 0 | 0 |
|  | 5 | 2 (0.2) | 0 | 2 (0.1) |
|  |  |  |  |  |
| Adverse Event | Total | 1 (0.1) | 0 | 1 (0.1) |
|  | 1 | 0 | 0 | 0 |
|  | 2 | 0 | 0 | 0 |
|  | 3 | 0 | 0 | 0 |
|  | 4 | 0 | 0 | 0 |
|  | 5 | 1 (0.1) | 0 | 1 (0.1) |
|  |  |  |  |  |
| Chills | Total | 1 (0.1) | 0 | 1 (0.1) |
|  | 1 | 1 (0.1) | 0 | 1 (0.1) |
|  | 2 | 0 | 0 | 0 |
|  | 3 | 0 | 0 | 0 |
|  | 4 | 0 | 0 | 0 |
|  | 5 | 0 | 0 | 0 |
|  |  |  |  |  |
| Multiple Organ Dysfunction Syndrome | Total | 0 | 1 (0.9) | 1 (0.1) |
|  | 1 | 0 | 0 | 0 |
|  | 2 | 0 | 0 | 0 |
|  | 3 | 0 | 0 | 0 |
|  | 4 | 0 | 0 | 0 |
|  | 5 | 0 | 1 (0.9) | 1 (0.1) |
|  |  |  |  |  |
| Infections And Infestations | Total | 5 (0.4) | 0 | 5 (0.4) |
|  | 1 | 1 (0.1) | 0 | 1 (0.1) |
|  | 2 | 1 (0.1) | 0 | 1 (0.1) |
|  | 3 | 2 (0.2) | 0 | 2 (0.1) |
|  | 4 | 0 | 0 | 0 |
|  | 5 | 1 (0.1) | 0 | 1 (0.1) |
|  |  |  |  |  |
| Bacterial Sepsis | Total | 1 (0.1) | 0 | 1 (0.1) |
|  | 1 | 0 | 0 | 0 |
|  | 2 | 0 | 0 | 0 |
|  | 3 | 1 (0.1) | 0 | 1 (0.1) |
|  | 4 | 0 | 0 | 0 |
|  | 5 | 0 | 0 | 0 |
|  |  |  |  |  |
| Gastroenteritis | Total | 1 (0.1) | 0 | 1 (0.1) |
|  | 1 | 0 | 0 | 0 |
|  | 2 | 1 (0.1) | 0 | 1 (0.1) |
|  | 3 | 0 | 0 | 0 |
|  | 4 | 0 | 0 | 0 |
|  | 5 | 0 | 0 | 0 |
|  |  |  |  |  |
| Respiratory Tract Infection | Total | 1 (0.1) | 0 | 1 (0.1) |
|  | 1 | 0 | 0 | 0 |
|  | 2 | 0 | 0 | 0 |
|  | 3 | 0 | 0 | 0 |
|  | 4 | 0 | 0 | 0 |
|  | 5 | 1 (0.1) | 0 | 1 (0.1) |
|  |  |  |  |  |
| Septic Shock | Total | 1 (0.1) | 0 | 1 (0.1) |
|  | 1 | 0 | 0 | 0 |
|  | 2 | 0 | 0 | 0 |
|  | 3 | 1 (0.1) | 0 | 1 (0.1) |
|  | 4 | 0 | 0 | 0 |
|  | 5 | 0 | 0 | 0 |
|  |  |  |  |  |
| Urinary Tract Infection | Total | 1 (0.1) | 0 | 1 (0.1) |
|  | 1 | 1 (0.1) | 0 | 1 (0.1) |
|  | 2 | 0 | 0 | 0 |
|  | 3 | 0 | 0 | 0 |
|  | 4 | 0 | 0 | 0 |
|  | 5 | 0 | 0 | 0 |
|  |  |  |  |  |
| Injury, Poisoning And Procedural Complications | Total | 4 (0.3) | 0 | 4 (0.3) |
|  | 1 | 1 (0.1) | 0 | 1 (0.1) |
|  | 2 | 0 | 0 | 0 |
|  | 3 | 3 (0.2) | 0 | 3 (0.2) |
|  | 4 | 0 | 0 | 0 |
|  | 5 | 0 | 0 | 0 |
|  |  |  |  |  |
| Infusion Related Reaction | Total | 4 (0.3) | 0 | 4 (0.3) |
|  | 1 | 1 (0.1) | 0 | 1 (0.1) |
|  | 2 | 0 | 0 | 0 |
|  | 3 | 3 (0.2) | 0 | 3 (0.2) |
|  | 4 | 0 | 0 | 0 |
|  | 5 | 0 | 0 | 0 |
|  |  |  |  |  |
| Metabolism And Nutrition Disorders | Total | 5 (0.4) | 0 | 5 (0.4) |
|  | 1 | 0 | 0 | 0 |
|  | 2 | 1 (0.1) | 0 | 1 (0.1) |
|  | 3 | 2 (0.2) | 0 | 2 (0.1) |
|  | 4 | 2 (0.2) | 0 | 2 (0.1) |
|  | 5 | 0 | 0 | 0 |
|  |  |  |  |  |
| Decreased Appetite | Total | 2 (0.2) | 0 | 2 (0.1) |
|  | 1 | 0 | 0 | 0 |
|  | 2 | 1 (0.1) | 0 | 1 (0.1) |
|  | 3 | 1 (0.1) | 0 | 1 (0.1) |
|  | 4 | 0 | 0 | 0 |
|  | 5 | 0 | 0 | 0 |
|  |  |  |  |  |
| Hyponatraemia | Total | 2 (0.2) | 0 | 2 (0.1) |
|  | 1 | 0 | 0 | 0 |
|  | 2 | 0 | 0 | 0 |
|  | 3 | 0 | 0 | 0 |
|  | 4 | 2 (0.2) | 0 | 2 (0.1) |
|  | 5 | 0 | 0 | 0 |
|  |  |  |  |  |
| Tumour Lysis Syndrome | Total | 1 (0.1) | 0 | 1 (0.1) |
|  | 1 | 0 | 0 | 0 |
|  | 2 | 0 | 0 | 0 |
|  | 3 | 1 (0.1) | 0 | 1 (0.1) |
|  | 4 | 0 | 0 | 0 |
|  | 5 | 0 | 0 | 0 |
|  |  |  |  |  |
| Musculoskeletal And Connective Tissue Disorders | Total | 1 (0.1) | 0 | 1 (0.1) |
|  | 1 | 0 | 0 | 0 |
|  | 2 | 1 (0.1) | 0 | 1 (0.1) |
|  | 3 | 0 | 0 | 0 |
|  | 4 | 0 | 0 | 0 |
|  | 5 | 0 | 0 | 0 |
|  |  |  |  |  |
| Muscular Weakness | Total | 1 (0.1) | 0 | 1 (0.1) |
|  | 1 | 0 | 0 | 0 |
|  | 2 | 1 (0.1) | 0 | 1 (0.1) |
|  | 3 | 0 | 0 | 0 |
|  | 4 | 0 | 0 | 0 |
|  | 5 | 0 | 0 | 0 |
|  |  |  |  |  |
| Nervous System Disorders | Total | 3 (0.2) | 0 | 3 (0.2) |
|  | 1 | 1 (0.1) | 0 | 1 (0.1) |
|  | 2 | 2 (0.2) | 0 | 2 (0.1) |
|  | 3 | 0 | 0 | 0 |
|  | 4 | 0 | 0 | 0 |
|  | 5 | 0 | 0 | 0 |
|  |  |  |  |  |
| Paraesthesia | Total | 2 (0.2) | 0 | 2 (0.1) |
|  | 1 | 0 | 0 | 0 |
|  | 2 | 2 (0.2) | 0 | 2 (0.1) |
|  | 3 | 0 | 0 | 0 |
|  | 4 | 0 | 0 | 0 |
|  | 5 | 0 | 0 | 0 |
|  |  |  |  |  |
| Headache | Total | 1 (0.1) | 0 | 1 (0.1) |
|  | 1 | 1 (0.1) | 0 | 1 (0.1) |
|  | 2 | 0 | 0 | 0 |
|  | 3 | 0 | 0 | 0 |
|  | 4 | 0 | 0 | 0 |
|  | 5 | 0 | 0 | 0 |
|  |  |  |  |  |
| Product Issues | Total | 1 (0.1) | 0 | 1 (0.1) |
|  | 1 | 0 | 0 | 0 |
|  | 2 | 1 (0.1) | 0 | 1 (0.1) |
|  | 3 | 0 | 0 | 0 |
|  | 4 | 0 | 0 | 0 |
|  | 5 | 0 | 0 | 0 |
|  |  |  |  |  |
| Device Leakage | Total | 1 (0.1) | 0 | 1 (0.1) |
|  | 1 | 0 | 0 | 0 |
|  | 2 | 1 (0.1) | 0 | 1 (0.1) |
|  | 3 | 0 | 0 | 0 |
|  | 4 | 0 | 0 | 0 |
|  | 5 | 0 | 0 | 0 |
|  |  |  |  |  |
| Psychiatric Disorders | Total | 1 (0.1) | 0 | 1 (0.1) |
|  | 1 | 0 | 0 | 0 |
|  | 2 | 1 (0.1) | 0 | 1 (0.1) |
|  | 3 | 0 | 0 | 0 |
|  | 4 | 0 | 0 | 0 |
|  | 5 | 0 | 0 | 0 |
|  |  |  |  |  |
| Insomnia | Total | 1 (0.1) | 0 | 1 (0.1) |
|  | 1 | 0 | 0 | 0 |
|  | 2 | 1 (0.1) | 0 | 1 (0.1) |
|  | 3 | 0 | 0 | 0 |
|  | 4 | 0 | 0 | 0 |
|  | 5 | 0 | 0 | 0 |
|  |  |  |  |  |
| Respiratory, Thoracic And Mediastinal Disorders | Total | 4 (0.3) | 1 (0.9) | 5 (0.4) |
|  | 1 | 4 (0.3) | 0 | 4 (0.3) |
|  | 2 | 0 | 0 | 0 |
|  | 3 | 0 | 1 (0.9) | 1 (0.1) |
|  | 4 | 0 | 0 | 0 |
|  | 5 | 0 | 0 | 0 |
|  |  |  |  |  |
| Cough | Total | 3 (0.2) | 0 | 3 (0.2) |
|  | 1 | 3 (0.2) | 0 | 3 (0.2) |
|  | 2 | 0 | 0 | 0 |
|  | 3 | 0 | 0 | 0 |
|  | 4 | 0 | 0 | 0 |
|  | 5 | 0 | 0 | 0 |
|  |  |  |  |  |
| Interstitial Lung Disease | Total | 0 | 1 (0.9) | 1 (0.1) |
|  | 1 | 0 | 0 | 0 |
|  | 2 | 0 | 0 | 0 |
|  | 3 | 0 | 1 (0.9) | 1 (0.1) |
|  | 4 | 0 | 0 | 0 |
|  | 5 | 0 | 0 | 0 |
|  |  |  |  |  |
| Oropharyngeal Pain | Total | 1 (0.1) | 0 | 1 (0.1) |
|  | 1 | 1 (0.1) | 0 | 1 (0.1) |
|  | 2 | 0 | 0 | 0 |
|  | 3 | 0 | 0 | 0 |
|  | 4 | 0 | 0 | 0 |
|  | 5 | 0 | 0 | 0 |
|  |  |  |  |  |
| Skin And Subcutaneous Tissue Disorders | Total | 4 (0.3) | 0 | 4 (0.3) |
|  | 1 | 0 | 0 | 0 |
|  | 2 | 4 (0.3) | 0 | 4 (0.3) |
|  | 3 | 0 | 0 | 0 |
|  | 4 | 0 | 0 | 0 |
|  | 5 | 0 | 0 | 0 |
|  |  |  |  |  |
| Pruritus Generalised | Total | 2 (0.2) | 0 | 2 (0.1) |
|  | 1 | 0 | 0 | 0 |
|  | 2 | 2 (0.2) | 0 | 2 (0.1) |
|  | 3 | 0 | 0 | 0 |
|  | 4 | 0 | 0 | 0 |
|  | 5 | 0 | 0 | 0 |
|  |  |  |  |  |
| Rash Erythematous | Total | 1 (0.1) | 0 | 1 (0.1) |
|  | 1 | 0 | 0 | 0 |
|  | 2 | 1 (0.1) | 0 | 1 (0.1) |
|  | 3 | 0 | 0 | 0 |
|  | 4 | 0 | 0 | 0 |
|  | 5 | 0 | 0 | 0 |
|  |  |  |  |  |
| Swelling Face | Total | 1 (0.1) | 0 | 1 (0.1) |
|  | 1 | 0 | 0 | 0 |
|  | 2 | 1 (0.1) | 0 | 1 (0.1) |
|  | 3 | 0 | 0 | 0 |
|  | 4 | 0 | 0 | 0 |
|  | 5 | 0 | 0 | 0 |
|  |  |  |  |  |
| Vascular Disorders | Total | 3 (0.2) | 0 | 3 (0.2) |
|  | 1 | 1 (0.1) | 0 | 1 (0.1) |
|  | 2 | 1 (0.1) | 0 | 1 (0.1) |
|  | 3 | 0 | 0 | 0 |
|  | 4 | 1 (0.1) | 0 | 1 (0.1) |
|  | 5 | 0 | 0 | 0 |
|  | | | | |
|  |  |  |  |  |
| Hypertension | Total | 1 (0.1) | 0 | 1 (0.1) |
|  | 1 | 0 | 0 | 0 |
|  | 2 | 0 | 0 | 0 |
|  | 3 | 0 | 0 | 0 |
|  | 4 | 1 (0.1) | 0 | 1 (0.1) |
|  | 5 | 0 | 0 | 0 |
|  |  |  |  |  |
| Hypotension | Total | 1 (0.1) | 0 | 1 (0.1) |
|  | 1 | 1 (0.1) | 0 | 1 (0.1) |
|  | 2 | 0 | 0 | 0 |
|  | 3 | 0 | 0 | 0 |
|  | 4 | 0 | 0 | 0 |
|  | 5 | 0 | 0 | 0 |
|  |  |  |  |  |
| Thrombophlebitis | Total | 1 (0.1) | 0 | 1 (0.1) |
|  | 1 | 0 | 0 | 0 |
|  | 2 | 1 (0.1) | 0 | 1 (0.1) |
|  | 3 | 0 | 0 | 0 |
|  | 4 | 0 | 0 | 0 |
|  | 5 | 0 | 0 | 0 |
|  |  |  |  |  |
| Percentages are calculated using number of subjects in each treatment group at baseline as a denominator. If a subject has two or more adverse events in the same system organ class (or with the same preferred term) with different Severity grades, then the event with the highest severity grade is used for that subject. System Organ Classes are sorted in alphabetical order. Preferred terms are sorted by descending order of frequency within System Organ Class. Adverse Events are coded using MedDRA Version 24.1 CTCAE (5.0) Severity Grade 1=Mild, 2=Moderate, 3=Severe, 4=Life threatening, 5=Death | | | | |
